# A Supervised Explainable Machine Learning Model for Perioperative Neurocognitive Disorder in Liver-Transplantation Patients and External Validation on the Medical Information Mart for Intensive Care IV Database: Retrospective Study

**DOI:** 10.2196/55046

**Published:** 2025-01-15

**Authors:** Zhendong Ding, Linan Zhang, Yihan Zhang, Jing Yang, Yuheng Luo, Mian Ge, Weifeng Yao, Ziqing Hei, Chaojin Chen

**Affiliations:** 1 Department of Anesthesiology The Third Affiliated Hospital of Sun Yat-sen University Guangzhou China; 2 Guangzhou AI & Data Cloud Technology Co., LTD Guangzhou China

**Keywords:** machine learning, risk factors, liver transplantation, perioperative neurocognitive disorders, MIMIC-Ⅳ database, external validation

## Abstract

**Background:**

Patients undergoing liver transplantation (LT) are at risk of perioperative neurocognitive dysfunction (PND), which significantly affects the patients’ prognosis.

**Objective:**

This study used machine learning (ML) algorithms with an aim to extract critical predictors and develop an ML model to predict PND among LT recipients.

**Methods:**

In this retrospective study, data from 958 patients who underwent LT between January 2015 and January 2020 were extracted from the Third Affiliated Hospital of Sun Yat-sen University. Six ML algorithms were used to predict post-LT PND, and model performance was evaluated using area under the receiver operating curve (AUC), accuracy, sensitivity, specificity, and *F*_1_-scores. The best-performing model was additionally validated using a temporal external dataset including 309 LT cases from February 2020 to August 2022, and an independent external dataset extracted from the Medical Information Mart for Intensive Care Ⅳ (MIMIC-Ⅳ) database including 325 patients.

**Results:**

In the development cohort, 201 out of 751 (33.5%) patients were diagnosed with PND. The logistic regression model achieved the highest AUC (0.799) in the internal validation set, with comparable AUC in the temporal external (0.826) and MIMIC-Ⅳ validation sets (0.72). The top 3 features contributing to post-LT PND diagnosis were the preoperative overt hepatic encephalopathy, platelet level, and postoperative sequential organ failure assessment score, as revealed by the Shapley additive explanations method.

**Conclusions:**

A real-time logistic regression model-based online predictor of post-LT PND was developed, providing a highly interoperable tool for use across medical institutions to support early risk stratification and decision making for the LT recipients.

## Introduction

Perioperative neurocognitive disorder (PND), encompassing various postsurgical cognitive impairments identified especially in the postoperative period, was first proposed in 2018 [1]. These cognitive changes are consistent with the clinical diagnostic criteria for neurocognitive disorders outlined in the *DSM-5* (*Diagnostic and Statistical Manual of Mental Disorders* [Fifth Edition]) [1-3]. In addition to postoperative delirium (POD) [4,5], other components of PND include emergence delirium, delayed neurocognitive recovery, and postoperative neurocognitive dysfunction [2,6]. POD or PND incidence is 2%-3% after general surgery [5,7] and 50%-70% in high-risk patients [8]. In addition, PND not only contributes to increased mortality rates but also extends hospitalization in patients undergoing liver transplantation (LT) [7,9], escalating health care costs and resource use. Preventative strategies and timely interventions for post-LT PND are crucial for enhancing patient outcomes and easing health care burdens [10].

Existing studies identify risk factors for post-LT PND, such as excessive alcohol consumption, Child-Turcotte-Pugh scores, and model for end-stage liver disease (MELD) scores [11,12]. Potential biomarkers for cognitive impairment prediction have also been proposed, including calcium binding protein β and neuron-specific enolase [13], yet their practical application is hindered by complex clinical scenarios and expense.

Machine learning (ML), a branch of artificial intelligence, offers a solution by distilling extensive clinical data into actionable insights, identifying relative risk factors for PND [14,15]. However, there is a dearth of ML-based models predicting post-LT–related complications [16-22] and postoperative delirium during specific surgeries [4,23]. There are currently no appropriate models for predicting PND in LT recipients, with most current clinical prediction models often failing to maintain accuracy when applied to external datasets, resulting in significant limitations to their generalizability.

This study aimed to extract critical predictors and develop an efficient ML algorithm to predict PND in LT recipients using routinely collected clinical data and to validate its performance using the Medical Information Mart for Intensive Care Ⅳ (MIMIC-Ⅳ) database.

## Material and Methods

### Study Design and Patients

This retrospective, single-center study was conducted at our institution following the Transparent Reporting of a multivariable prediction model for Individual Prognosis or Diagnosis guidelines. We enrolled 1267 patients who underwent LT between January 2015 and August 2022. Records were extracted using the perioperative specialist database platform (PSDP) and electronic patient record (EPR) systems. The inclusion and exclusion criteria are shown in Textbox 1.

All included recipients were formalized and registered in the China Organ Transplant Response System.

**Inclusion and exclusion criteria for the study.**

**Inclusion criteria**

- Age >18 years.
- Allogeneic liver transplantation.

**Exclusion criteria**

- Simultaneous liver and kidney transplantation.
- Preoperative overt hepatic encephalopathy.
- Emergency reoperation.
- Persistent postoperative coma and inability to screen for cognitive function.
- Post–liver transplantation cerebral infarction or hemorrhage.
- Incomplete medical records.

### Data Collection

The development and temporal validation cohort datasets were created by extracting original records from the Docare System (Medical system), Hospital Information System, and Laboratory Information System, and integrating them into the PSDP platform and EPR systems. To increase ML model accuracy and applicability, we included the following variables: (1) demographic characteristics; (2) liver donor characteristics; (3) preoperative comorbidities, complications, preoperative treatment, and LT etiology; (4) preoperative laboratory test results; (5) intraoperative surgery characteristics and medications; (6) postoperative MELD scores, sequential organ failure assessment (SOFA) scores, and laboratory test results; and (7) complications and prognosis in LT recipients. All of the original data were made anonymous throughout the study.

### Definitions of Outcomes

The primary outcome was postoperative PND occurrence from surgery until discharge from the hospital. A summary of perioperative neurocognitive impairments is shown in Table S1 in Multimedia Appendix 1**.** The initial diagnosis criteria was the retrieval of any of the following terms from the medical records: “Delirium”, “Confusion”, “Confusional arousals”, “Clouding of consciousness”, “Soma”, “Drowsiness”, “Changes in mental status”, “Hallucinations”, “Disorientation”, “Dyscalculia”, “Haziness of spirit-mind”, “Irritability”, “Agitation”, “Inattentiveness”, “Reactive confusion”, “Somatization disorder”, “Irritability”, and “Somatoform disorders”, or equivalent terms in Chinese [4,24,25]. Next, each patient was evaluated based on the *DSM-5* criteria by a designated neurologist without prior access to the patient’s records [3,26].

### Variable Selection

A comprehensive set of 137 variables was extracted for the initial analysis (Table S2 in Multimedia Appendix 1). Table S3 in Multimedia Appendix 1 provides a concise explanation of the main complications and relevant term definitions. Postoperative SOFA scores were calculated by intensive care unit (ICU) physicians immediately after surgery according to European Society of Intensive Care Medicine criteria [27] and submitted for statistical analysis.

To account for multicollinearity and confounding variables affecting the overall model fitting performance, variables that were statistically significant (*P*<.05) in the univariate test were subjected to stability selection (Table S4 in Multimedia Appendix 1) [28]. After 100 iterations of least absolute shrinkage and selection operator (LASSO) regression, the top 10 features with the highest selection frequencies were chosen to train the ML models. For each LASSO regression, 90% of the training set samples were randomly selected as subsamples.

### Machine Learning Models

The following 6 ML models were developed, and their performances were further evaluated: logistic regression (LR), multilayer perceptron classifier (MLP), extreme gradient boosting with classification trees (XGB), light gradient boosting machine (LGB), support vector machine (SVM), and random forest classifier (RF). All models were constructed using the XGB, LGB, and Scikit-learn packages.

The primary cohort dataset was randomly divided into 80% development and 20% internal validation sets. The bootstrap method was implemented 1000 times on the internal validation set to determine a 95% CI for the discrimination assessment metrics for each model: the area under the receiver operating curve (AUC), accuracy, sensitivity, specificity, and *F*_1_-scores. Considering that ML models have multiple hyperparameters that are essential for model performance, a 5-fold cross-validation grid search method was used to optimize the parameters and AUCs (Table S5 in Multimedia Appendix 1). The Shapley additive explanations (SHAP) method was used to assess predictive feature importance and explain the ML algorithms’ predictions [29].

### Model Performance Comparison and MIMIC-Ⅳ Dataset

Because the SOFA and MELD scores have been reported as potential predictors of various post-LT complications [16,30], our study also compared the ML model’s performance against SOFA and MELD scores.

An external validation set extracted from the MIMIC-Ⅳ (version 2.2) [31] database was used to evaluate the ML model’s performance, which was authorized by the review committee of Massachusetts Institute of Technology (agreement 1.5.0). Patients who underwent LT surgery and were diagnosed with PND according to the International Classification of Diseases (9th and 10th revisions) were enrolled. Data extraction and cleaning were performed using PostgreSQL (version 15.3) and Navicate Premium (version 16) with a Structured Query Language (Figure S1 in Multimedia Appendix 1).

### Statistical Analysis

Data cleaning used Python (version 3.9.13) packages Pandas (version 1.4.4) and Numpy (version 1.23.5). Data analysis used the Python Scipy package (version 3.7), and SHAP (0.41.0) was used to visualize and analyze feature importance.

Data distribution was evaluated using the Kolmogorov Smirnov test. Normally distributed continuous variables are presented as mean (SD) and were compared by independent sample *t* tests. Non-normally distributed continuous data are presented as median (IQR) and were compared using the nonparametric equivalent (Mann Whitney test). Categorical variables are expressed as frequencies and percentages and were tested using the chi-square test or Fisher exact test. Long-term survival rates were estimated using the Kaplan Meier method. Group comparisons were conducted using the Gehan-Breslow Wilcoxon test and log-rank tests.

All tests were 2-tailed, with statistical significance set at 0.05. Before ML model training, continuous variables were normalized, dichotomous variables were coded as binary variables, and multicategory variables were coded as uniform numbers.

Variables with missing values exceeding 20% were excluded, and missing values below 20% were imputed with the median (for numeric variables) or mode (for categorical variables). The overall data distribution after imputation exhibited an acceptable level of variability.

### Visualized Online Calculator

An online calculator with a visual interface was developed to facilitate the easy input of clinical variables and to generate clear and meaningful output indicating the absolute risk in percentages.

### Ethical Considerations

The study protocol was approved by the Ethics Committee of the Third Affiliated Hospital of Sun Yat-sen University on July 27, 2022 (No. (2019)02-609-04) and was conducted in accordance with the Declaration of Helsinki. The requirement for informed patient consent was waived due to the study’s retrospective nature, and all data were anonymized before analysis.

## Results

### Patient Demographic Characteristics

**Figure 1:**
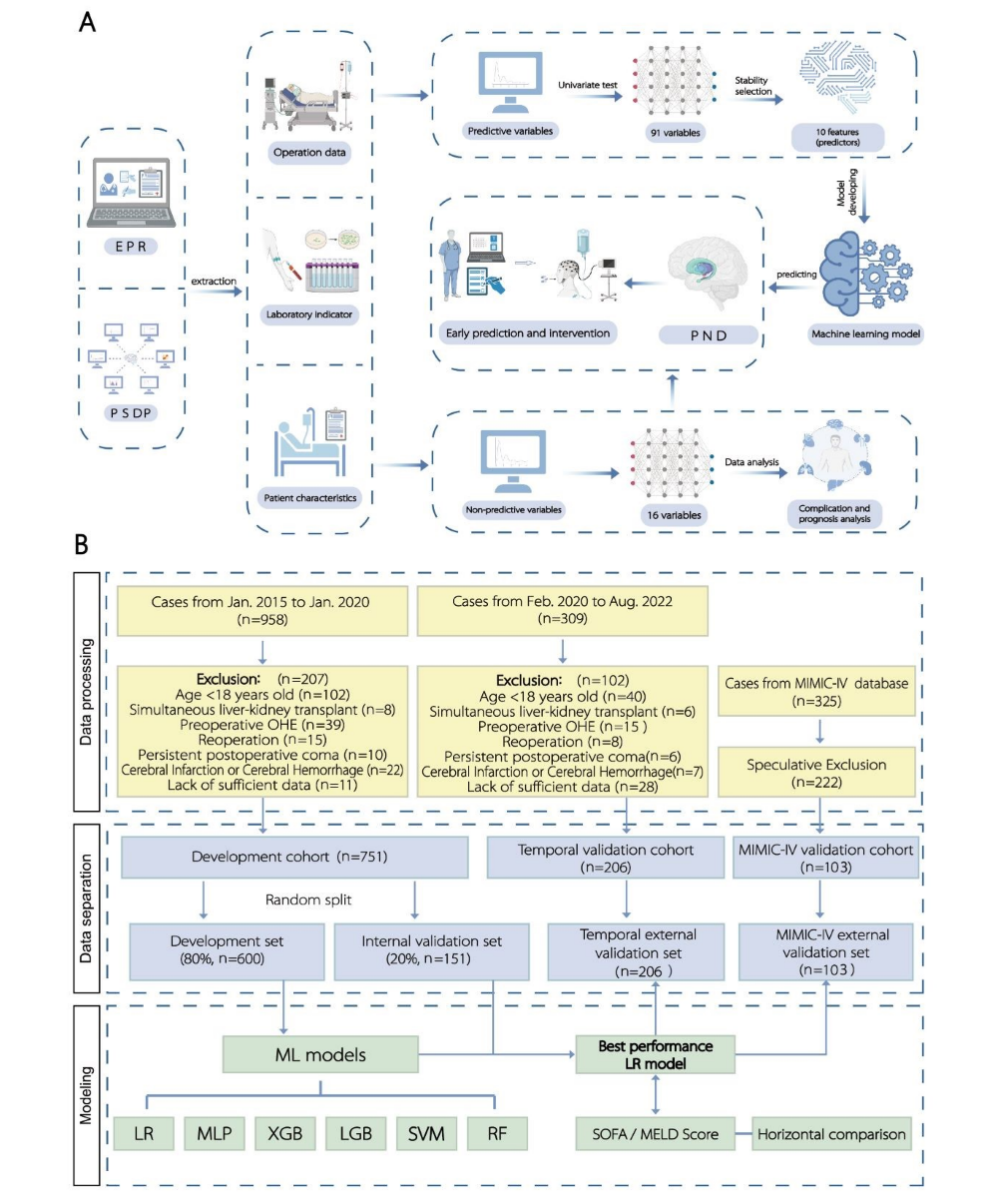
Diagram of experimental procedure and flowchart, (A) brief diagram of the experimental procedure and (B) flowchart for patient enrollment, development and selection of machine learning model. LGB: light gradient boosting machine; LR: logistic regression; MIMIC-Ⅳ: the Medical Information Mart for Intensive Care Ⅳ; ML: machine learning; MLP: multilayer perceptron classifier; RF: random forest classifier; SVM: support vector machine; XGB: extreme gradient boosting with classification trees.

The flowchart for patient recruitment is shown in Figure 1. Of the 958 patients who underwent LT, 751 patients were enrolled randomly into the development set (n=600) and internal validation set (n=151). Notably, PND occurred in 201 patients, accounting for 33.5% of the development cohort. Table 1 and Table S6 in Multimedia Appendix 1 summarizes the development set’s demographic characteristics, donor features, and perioperative variables of patients with or without post-LT PND.

**Table 1:** Demographic characteristics and donor characteristics variables of patients with stratification by perioperative neurocognitive disorder.^a^

Characteristics			Total (n=600)	NonPND^b^ (n=399)	PND^b^ (n=201)	*P* value	
**Demographic characteristics**							
	Age (years), mean (SD)		49 (10.34)	49.24 (10.16)	48.53 (10.7)	.43	
	**Sex**						.06
		Female, n (%)	74 (12.33%)	42 (10.63%)	32 (16%)		
		Male, n (%)	521 (86.83%)	353 (89.37%)	168 (84%)		
	Height (cm), median (IQR)		170 (172-165)	170 (172-165)	169 (170-163)	.17	
	Weight (kg), median (IQR)		64 (71-58)	64 (72-58.88)	64 (70-58)	.26	
	BMI, median (IQR)		22.84 (24.85-20.43)	22.78 (24.90-20.45)	22.86 (24.74-20.44)	.79	
	**Blood group, n (%)**						.76
		A	233 (38.83)	156 (39.49)	77 (38.5)		
		B	156 (26)	106 (26.84)	50 (25)		
		O	167 (27.83)	110 (27.85)	57 (28.5)		
		AB	39 (6.5)	23 (5.82)	16 (8)		
**Donor characteristics**							
	Donor age (years), median (IQR)		40 (49-28)	40 (50-28)	40 (48-29.5)	.64	
	Donor BMI, median (IQR)		22.49 (24.22-20.7)	22.49 (24.22-20.76)	22.59 (24.52-20.38)	.35	
	**Donor Type**						.03
		DBD^c^, n (%)	321 (53.5)	228 (62.3)	93 (50.54)		
		DCD^d^, n (%)	225 (37.5)	136 (37.16)	89 (48.37)		
		DBCD^e^, n (%)	4 (0.67)	2 (0.55)	2 (1.09)		
	**Steatosis of donor liver**						.27
		Steatosis grade 0, n (%)	390 (65)	265 (71.05)	125 (65.79)		
		Steatosis grade 1, n (%)	147 (24.5)	94 (25.2)	53 (27.89)		
		Steatosis grade 2, n (%)	26 (4.33)	14 (3.75)	12 (6.32)		

^a^Data presented in Table 1 are based on the original dataset prior to data imputation.

^b^PND: perioperative neurocognitive dysfunction.

^c^DBD: donation after brain death.

^d^DCD: donation after circulatory death.

^e^DBCD: donation after brain death followed by circulatory death.

### Perioperative Characteristics

Among the preoperative characteristics, American Society of Anesthesiologists classification and preoperative comorbidities such as acute respiratory distress syndrome, and laboratory results including hemoglobin, white blood cell (WBC) count, liver function, coagulation function, and serum calcium were significantly different between patients with and without postoperative PND (*P*<.01, Table S6 in Multimedia Appendix 1). Specifically, individuals diagnosed with post-LT PND exhibited a notably elevated prevalence of preoperative cover hepatic encephalopathy (CHE; 45.27% vs 8.77%, *P*<.001) and hypercalcemia (7.57% vs 1.36%, *P*<.001). Furthermore, patients with post-LT PND had higher Child Pugh and MELD scores (*P*<.001), longer preoperative ICU stays, increased continuous blood purification, increased plasma exchange, longer mechanical ventilation, and higher tracheal intubation (all *P*<.001, Table S6 in Multimedia Appendix 1).

Regarding intraoperative characteristics, patients with post-LT PND had longer anesthesia durations; increased sodium bicarbonate levels, red blood cell counts, plasma levels, and levels of cryoprecipitate transfusion; increased estimated blood loss (EBL); and reduced urine output (all *P*<.001, Table S6 in Multimedia Appendix 1). Differences in intraoperative medications between the 2 groups were not significant, except for recombinant activated factor VII (*P*<.001). Interestingly, our results showed no association between day or night surgery and the incidence of PND (*P=*.44, Table S6 in Multimedia Appendix 1).

For the postoperative characteristics, patients with post-LT PND showed significantly higher levels of aspartate aminotransferase (AST), total bilirubin, blood urea nitrogen, prothrombin time (PT), international normalized ratio, hypersensitive C-reactive protein (hsCRP), procalcitonin, and serum calcium, as well as lower levels of hemoglobin, hematocrit, WBC, platelet (PLT), gamma-glutamyltransferase, albumin, and serum osmolality (all *P*<.05, Table S6 in Multimedia Appendix 1).

### Feature Selection

The frequency of LASSO algorithm selection for each variable is shown in detail in Figure S2 in Multimedia Appendix 1. The top 10 features chosen as predictors for ML model development were preoperative CHE, PLT, PT, estimated glomerular filtration rate (eGFR), Ca^2+^, MELD score, intraoperative EBL, postoperative SOFA score, hsCRP, and AST.

### Model Performance and Horizontal Comparison

**Figure 2:**
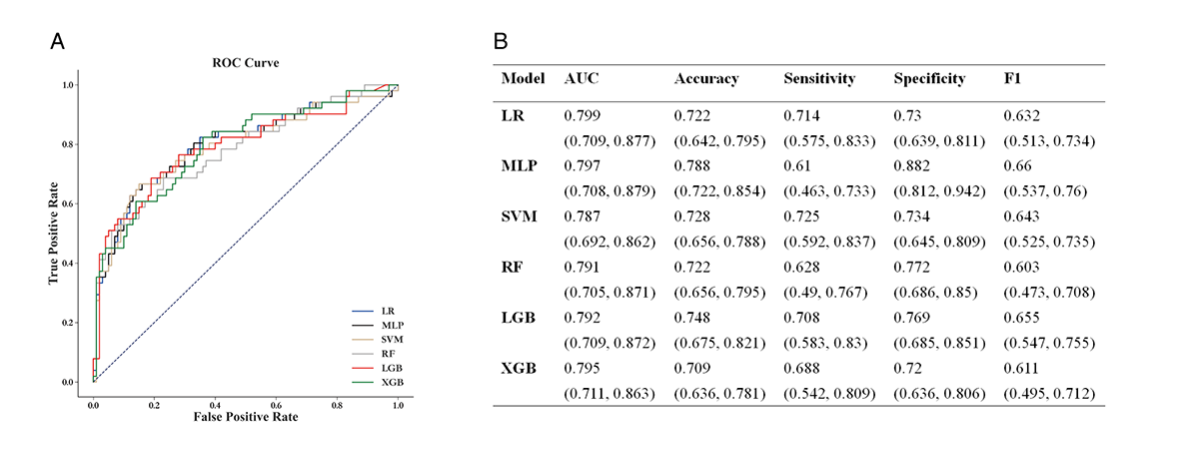
Performance metrics for six ML models. (A) ROC curves of six ML models. (B) Details of the model performance metrics. Accuracy=(TP+TN)/(TP+TN+FP+FN); AUC, the area under the receiver-operating curve; F1=2*Precision*Recall/ (Precision + Recall); FN: false negative; FP: false positive; LGB: light gradient boosting machine; LR: logistic regression; MLP: multilayer perceptron classifier; RF: random forest classifier; Sensitivity=TP/ (TP + FN); Specificity (Recall)=TN/ (TN + FP); SVM: support vector machine; TN: true negative; TP: true positive; XGB: extreme gradient boosting with classification trees.

The performance of the 6 ML models is shown in Figure 2. The LR model achieved the highest AUC (0.799, 95% CI 0.709-0.877) with acceptable accuracy (0.722, 95% CI 0.642-0.795), sensitivity (0.714, 95% CI 0.575-0.833), and specificity (0.73, 95% CI 0.639-0.811) compared with the other 5 models.

**Figure 3:**
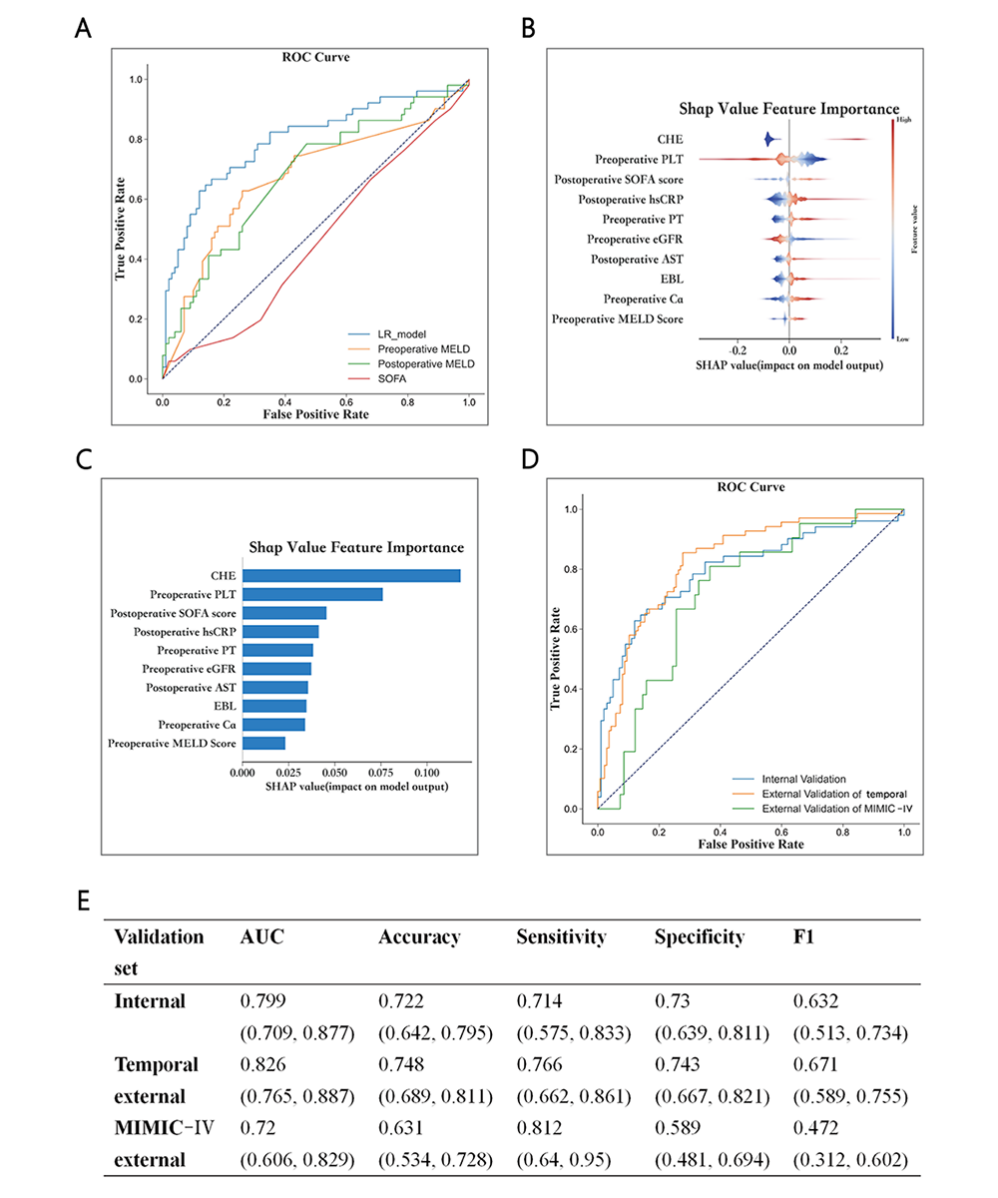
SHAP analysis of the LR model and model performance in horizontal comparison and external validation. (A) Horizontal comparison of predicting performance between the LR model and MELD/SOFA scores in the internal validation set. (B-C) The SHAP summary plot demonstrated the general importance of each feature in LR model. The color bar on the right indicates the relative value of a feature in each case, with red color representing higher value and blue color representing lower value. (D-E) ROC curves and model performance in the external validation. AST: aspartate aminotransferase; AUC: the area under the receiver-operating curve; CHE: cover hepatic encephalopathy; EBL: estimated blood loss; eGFR: estimated glomerular filtration rate; hsCRP: hypersensitive C-reactive protein; LR: logistic regression; MELD scores: model for end-stage liver disease score; MIMIC-IV: Medical Information Mart for Intensive Care Ⅳ; PLT: platelet; PT: prothrombin time; SHAP: Shapley additive explanations; SOFA scores: sequential organ failure assessment score.

The SOFA (AUC=0.459, 95% CI 0.365-0.555), preoperative MELD (AUC=0.672, 95% CI 0.581-0.768), and postoperative MELD scores (AUC=0.679, 95% CI 0.587-0.772) had significantly lower AUCs than the LR model in the internal validation set (Figure 3A).

### Feature Importance

The SHAP summary plot (Figures 3B and 3C) illustrates the correlation between the feature value magnitudes in the LR model. Both SHAP plots revealed that the presence of CHE, lower preoperative PLT, higher postoperative SOFA score, higher postoperative hsCRP, and higher preoperative PT were associated with a higher SHAP value output in the LR model, indicating a heightened likelihood of post-LT PND and forming the top 5 effective variables.

Three correctly classified examples (eg, patients 48, 80, and 122) are presented in Figure S3 in Multimedia Appendix 1, showing the SHAP decision and force plots.

### Temporal External Validation and MIMIC-Ⅳ Dataset Validation

A comparison of the main demographic characteristics and key predictive variables between the development and validation sets is shown in Table S7 in Multimedia Appendix 1, and the incidence rates of post-LT PND in the temporal and MIMIC-Ⅳ external validation were 27.1%, and 20.3%, respectively. The LR model exhibited a comparable performance in the temporal external validation set (AUC=0.826, 95% CI 0.765-0.887) (Figure 3D). Surprisingly, the LR model also provided acceptable predictions for the MIMIC-Ⅳ dataset (Figure 3D, AUC=0.72, 95% CI 0.606-0.829). Figure 3E summarizes the main performance metrics of the LR model.

### Effect of Perioperative Neurocognitive Dysfunction on Patients’ Outcomes and Prognosis

Compared with patients without post-LT PND, patients with PND were more likely to experience perioperative complications (Table S8 in Multimedia Appendix 1), including higher incidences of sepsis (51.63% vs 21.55%, *P*<.001), pneumonia (75.56% vs 65.46%, *P*<.05), acute kidney injury (69.5% vs 39.75%, *P*<.001), and hemodialysis (51.35% vs 12.81%, *P*<.001). Furthermore, patients with post-LT PND had higher hospitalization costs (CNY 377,801.69 [US $51,566.83], SD 177,855.53 [US $24,275.82] vs CNY 277,018.95 [US $37,810.82], SD 92,779.91 [US $12,663.70]; *P*<.001), prolonged postoperative stays (25 {18} vs 21 {11} days, *P*<.001), longer postoperative ICU stay (113 {114} vs 65 {48.5} hours, *P*<.001), and a markedly higher in-hospital mortality rate (12.44% vs 2.51%, *P*<.001).

**Figure 4:**
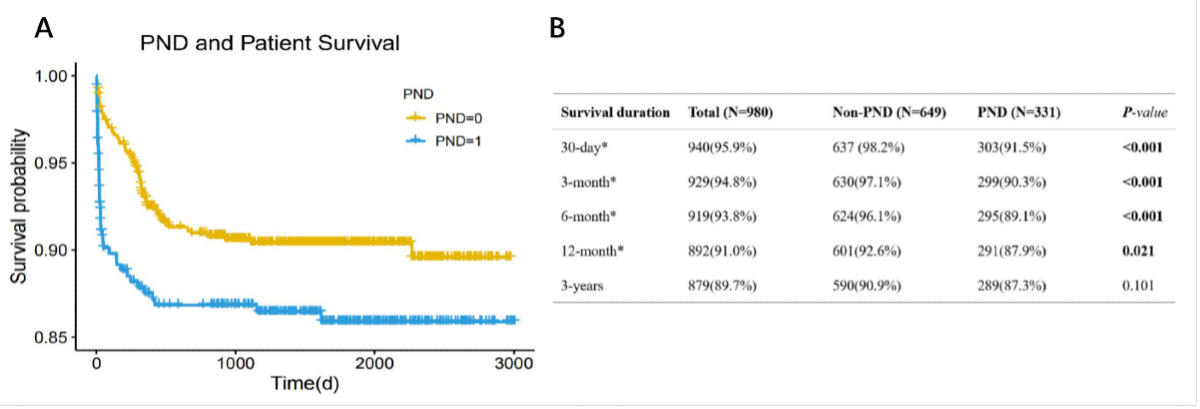
Post–liver transplantation survival associated with perioperative neurocognitive dysfunction. Patients with post–liver transplantation perioperative neurocognitive dysfunction showed a significantly lower survival rate. LT: liver transplantation; PND: perioperative neurocognitive dysfunction.

Further survival analysis (Figure 4) was conducted to assess patient prognosis. The PND group exhibited significantly lower survival rates at 30 days (91.5% vs 98.2%, *P*<.001), 3 months (90.3% vs 97.1%, *P*<.001), 6 months (89.1% vs 96.1%, *P*<.001), and 12 months (87.9% vs 92.6%, *P*=.02).

### Clinical Availability of the Logistic Regression Model

**Figure 5:**
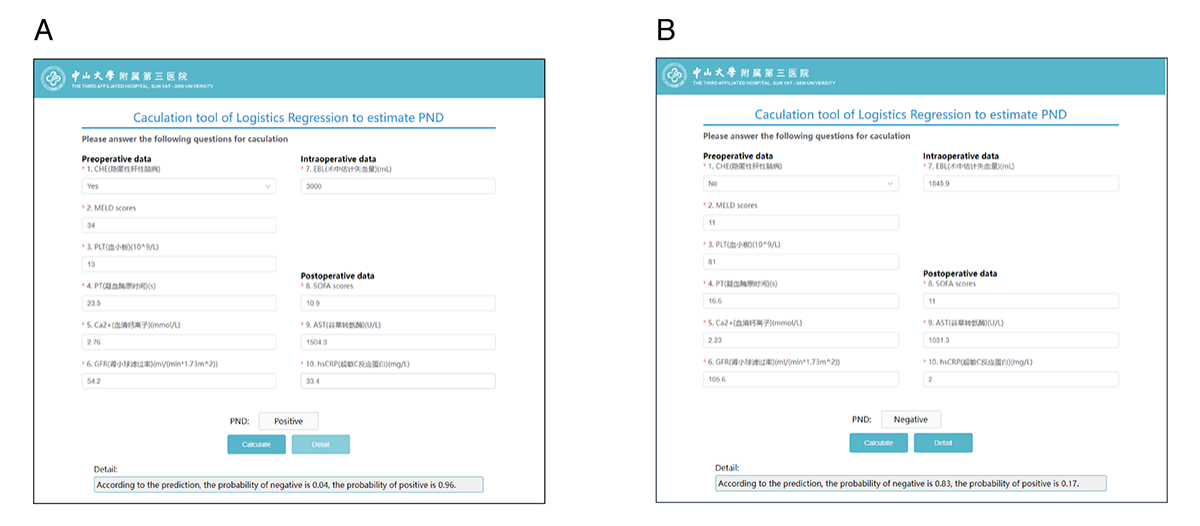
Online calculator for the clinical interface of the post–liver transplantation perioperative neurocognitive dysfunction risk prediction logistic regression model. (A) Patient No. 48 post–liver transplantation perioperative neurocognitive dysfunction will occur (probability of perioperative neurocognitive dysfunction: 94%); (B) Patient No. 122 post–liver transplantation perioperative neurocognitive dysfunction will not occur (probability of perioperative neurocognitive dysfunction: 17%).

Given the accessibility of the 10 predictive features, we constructed a visually oriented online calculator to facilitate clinical decision making. The perioperative information of 2 typical patients was entered into the online calculator: patient 48 had a positive final predicted probability of PND occurrence (probability: 96%), and patient 122 had a negative final predicted probability of PND occurrence (probability: 17%; Figure 5). The online calculator is freely accessible at the hospital website.

## Discussion

### Principal Findings

Our retrospective study assessed 6 different ML algorithms to predict post-LT PND, using 10 readily available clinical parameters. We found that post-LT PND incidence was 33.5%. The 10 predictive features significantly associated with PND included preoperative CHE, PLT, PT, eGFR, Ca^2+^, MELD score, intraoperative EBL and postoperative SOFA score, hsCRP, and AST. The LR model demonstrated superior performance, with high AUC, accuracy, sensitivity, and specificity, surpassing traditional SOFA and MELD scores in predicting post-LT PND and performed acceptably in the rigorous temporal and MIMIC-Ⅳ external validations.

This study aids clinicians in detecting postoperative cognitive changes in LT recipients. Patients with PND typically faced more perioperative complications, higher hospitalization costs, and prolonged hospital and ICU stays, consistent with previous studies [4,23]. Hepatic encephalopathy has been reported as an independent risk factor for postoperative neurocognitive disorders [32]. To ensure cognitive assessment accuracy, we excluded patients with overt hepatic encephalopathy according to the spectrum of neurocognitive impairment in cirrhosis criteria [33]. CHE emerged as a significant predictor in our model analysis. Both oxidative stress and neuroinflammation have been implicated in POD pathophysiology [10,34]. A recent systematic review also links increased perioperative CRP levels to a high delirium risk [35], supporting our inclusion of hsCRP as a predictor. Calcium ions (Ca^2+^) are important cell signaling molecules, and previous studies reported a positive correlation between Ca^2+^ concentration and neuronal apoptosis extent in vitro [36]*,* consistent with our results. Furthermore, the model identified PLT as an unconventional indicator of PND, showcasing ML’s ability to highlight nontraditional risk factors. This discovery is partly supported by Eyer et al [37] suggesting a relationship between lower PLT and delirium tremens.

Our study used preoperative, intraoperative, and postoperative data (SOFA scores, hsCRP, and AST levels) to develop the LR model. Earlier studies have revealed that multiple postoperative factors were also risk factors for PND [11,12]. The postoperative variables included in this study were predominantly assessed upon initial admission to the ICU. Stability selection analysis revealed a positive correlation between elevated postoperative SOFA scores, hsCRP levels, and AST levels, and an increased likelihood of post-LT PND. This highlights the predictive value of these commonly observed postoperative variables for PND.

Our results suggest that LR outperforms other ML models in predicting post-LT PND, which is not surprising. A recent systematic review showed no performance superiority of other ML models over LR in predicting clinical complications [38]. Wiredu et al [35] also found that compared to ML algorithms, LR had the highest AUC when predicting sex-specific hip fractures. Song et al [4] developed an LR model to predict POD in older adult patients, achieving the highest AUC compared with other models. Given the evident linear relationships among the top 10 features, the LR may be more appropriate for capturing distribution patterns. In contrast to other algorithms, LR performs well on nonoversized and high-dimensional datasets, exhibits computational efficiency, and imposes lower dataset requirements.

As demonstrated by the example of prediction cases (Figure 5), we successfully developed a predictive model for post-LT PND, with its primary advantage in its reliable predictive performance, validated using 2 external datasets. The importance of early detection and prevention of PND in patients undergoing cardiac surgery or transplantation is clearly emphasized in current international guidelines [39]. However, the implementation of preventive measures is often challenged by limited resources [40], especially in cases where the shortage of liver donors persists. On accurate identification by the LR model, patients at high risk for post-LT POD could be referred to enhanced LT perioperative management strategies, such as individualized pharmacological or nonpharmacological comprehensive multicomponent interventions, according to the 10 commonly accessible predictive parameters filtered by the ML algorithm.

### Limitations

However, this study had several limitations. First, it was a single-center retrospective study, meaning the Confusion Assessment Method (CAM) or the associated CAM-ICU and 3D-CAM were inappropriate for our database. Instead, patients with PND were identified from medical records according to the *DSM-5* criteria [3,6,26]. Second, as a real-world study, researchers can only infer precise risk factors based on the data available, and inhomogeneous confounding among the datasets could affect the study conclusions [41]. While our online decision tool has the potential to aid surgeons and anesthesiologists in clinical decision making, the causes and underlying mechanisms of PND remain subjects of intense debate, necessitating further research.

### Conclusions

This study successfully develops a real-time and easily accessible parameter requiring LR-based PND prediction algorithm for post-LT settings. The LR model outperformed the other five models owing to its enhanced model performance and interpretability. The optimal use of our freely accessible online predictor would enable timely and convenient risk stratification, enhanced perioperative management strategies, and comprehensive multicomponent interventions.

## Appendix

Multimedia Appendix 1 Additional online content.

## Abbreviations

AST: aspartate aminotransferase
AUC: area under the receiver operating curve
CAM: Confusion Assessment Method
CHE: cover hepatic encephalopathy
DSM-5: Diagnostic and Statistical Manual of Mental Disorders (Fifth Edition)
EBL: estimated blood loss
eGFR: estimated glomerular filtration rate
EPR: electronic patient record
hsCRP: hypersensitive C-reactive protein
ICU: intensive care unit
LASSO: least absolute shrinkage and selection operator
LGB: light gradient boosting machine
LR: logistic regression
LT: liver transplantation
MELD: model for end-stage liver disease
MIMIC-Ⅳ: Medical Information Mart for Intensive Care Ⅳ
ML: machine learning
MLP: multilayer perceptron classifier
PLT: platelet
PND: perioperative neurocognitive disorder
POD: postoperative delirium
PSDP: perioperative specialist database platform
PT: prothrombin time
RF: random forest classifier
SHAP: Shapley additive explanations
SOFA: sequential organ failure assessment
SVM: support vector machine
WBC: white blood cell
XGB: extreme gradient boosting with classification trees

## Data Availability

All data generated or analyzed during this study are included in this published article and Multimedia Appendix 1, and the codes used in this study are all common codes in Python packages mentioned in the “Methods” section of the manuscript.
