# Correction: A Supervised Explainable Machine Learning Model for Perioperative Neurocognitive Disorder in Liver-Transplantation Patients and External Validation on the Medical Information Mart for Intensive Care IV Database: Retrospective Study

**DOI:** 10.2196/88952

**Published:** 2026-03-11

**Authors:** Zhendong Ding, Linan Zhang, Yihan Zhang, Jing Yang, Yuheng Luo, Mian Ge, Weifeng Yao, Ziqing Hei, Chaojin Chen

**Affiliations:** 1 Department of Anesthesiology The Third Affiliated Hospital of Sun Yat-sen University Guangzhou, Guangdong China; 2 Guangzhou AI & Data Cloud Technology Co., LTD Guangzhou China

In “A Supervised Explainable Machine Learning Model for Perioperative Neurocognitive Disorder in Liver-Transplantation Patients and External Validation on the Medical Information Mart for Intensive Care IV Database: Retrospective Study” [1], the authors made three corrections.

The footnotes of Table 1 originally read as follows:

^a^PND: perioperative neurocognitive dysfunction.

^b^DBD: donation after brain death.

^c^DCD: donation after circulatory death.>

^d^DBCD: donation after brain death followed by circulatory death.

The footnotes now read:

^a^Data presented in Table 1 are based on the original dataset prior to data imputation.

^b^PND: perioperative neurocognitive dysfunction.

^c^DBD: donation after brain death.

^d^DCD: donation after circulatory death.

^e^DBCD: donation after brain death followed by circulatory death.

Below is the new Figure 4 (presented here as Figure 1), which will replace the originally published figure.

**Figure 1:**
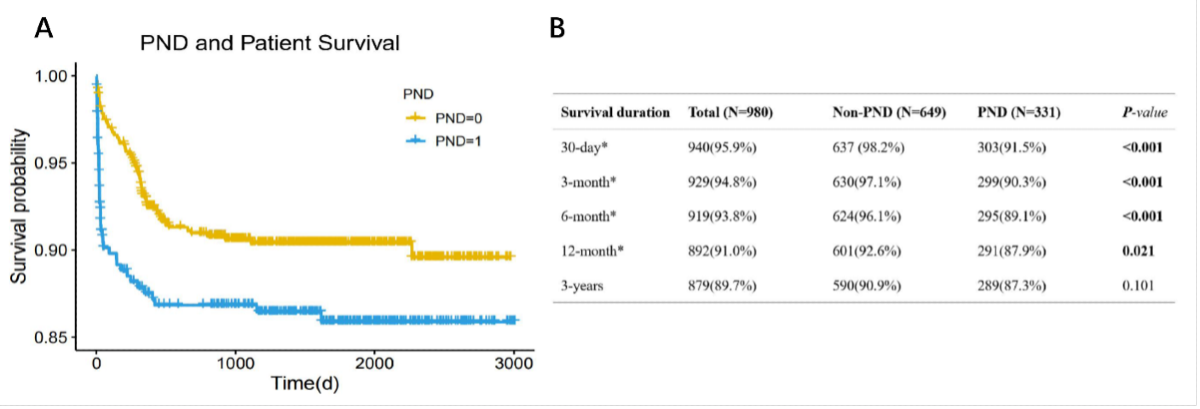
Post–liver transplantation survival associated with perioperative neurocognitive dysfunction. Patients with post–liver transplantation perioperative neurocognitive dysfunction showed a significantly lower survival rate. LT: liver transplantation; PND: perioperative neurocognitive dysfunction.

A paragraph in the *Effect of PND on Patients’ Outcomes and Prognosis* section has been revised. The text originally read as follows:

Further survival analysis (Figure 4)was conducted to assess patient prognosis. The PND group exhibited significantly lower survival rates at 30 days (87.1% vs 97.84%, *P*<.001), 3 months (83.99% vs 96.46%, *P*<.001), 6 months (82.78% vs 95.38%, *P*<.001), and 12 months (78.85% vs 88.44%, *P*<.001), and overall survival (*P*=.03).

The text now reads:

*Further survival analysis (Figure 4) was conducted to assess patient prognosis. The PND group exhibited significantly lower survival rates at 30 days (91.5% vs 98.2%, *P*<.001), 3 months (90.3% vs 97.1%, *P*<.001), 6 months (89.1% vs 96.1%, *P*<.001), and 12 months (87.9% vs 92.6%, *P*=.02).*

The correction will appear in the online version of the paper on the JMIR Publications website, together with the publication of this correction notice. Because this was made after submission to PubMed, PubMed Central, and other full-text repositories, the corrected article has also been resubmitted to those repositories.
