# Supplementary material for: A Supervised Explainable Machine Learning Model for Perioperative Neurocognitive Disorder in Liver-Transplantation Patients and External Validation on the Medical Information Mart for Intensive Care IV Database: Retrospective Study
Source: J Med Internet Res. 2025 Jan 15;27:e55046. doi: 10.2196/55046 (PMC11780294; doi:10.2196/55046)
Supplement: Multimedia Appendix 1 [file jmir_v27i1e55046_app1.docx]

**Supplementary Online Content**

**A Supervised Explainable Machine Learning Model for Perioperative Neurocognitive Disorder in Liver-Transplantation Patients: Retrospective Study With External Validation on the Medical Information Mart for Intensive Care Ⅳ Database**

**Supplemental Figure 1** Flow chart for patients’ enrollment from MIMIC-Ⅳ database.

**Supplemental Figure 2** All variables selected from perioperative dataset.

**Supplemental Figure 3** Examples of SHAP decision plot and force plot.

**Supplemental Table 1** A summary of the new nomenclature of perioperative neurocognitive impairments.

**Supplemental Table 2** All variables selected for initial selection.

**Supplemental Table 3** A summary of the definition of the main complications or relative terms in our study according to our previous study.

**Supplemental Table 4** All variables selected by univariate test.

**Supplemental Table 5** Details of the optimal hyperparameters for each classifier.

**Supplemental Table 6**. Perioperative variables of patients with stratification by perioperative neurocognitive disorder.

**Supplemental Table 7** The comparison of the main demographic characteristics and key predicting variables between the development set and validation set

**Supplemental Table 8** Comparison of complications and prognosis between Non-PND group and PND group.

**Supplemental Figure 1** Flow chart for patients’ enrollment from MIMIC-Ⅳ database.


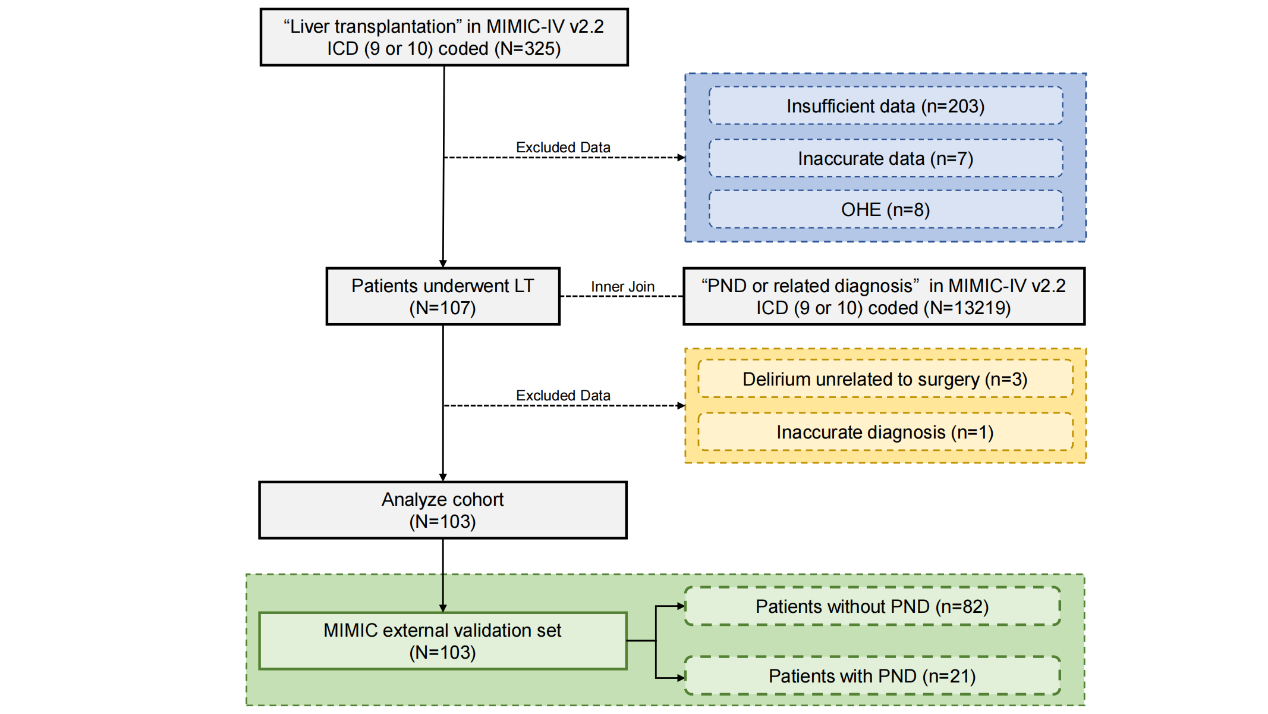


Note: The predictive variable hsCRP was insufficient in the MIMIC database, to complete the speculative external validation of this study, we extracted CRP as a surrogate. Speculative Diagnosis of Hepatic Encephalopathy: OHE: “HE” + “GCS<13”; CHE: “HE” + “GCS≥13”.

Abbreviations: OHE, overt hepatic encephalopathy; PND, perioperative neurocognitive disorder; ICD, international code designator.

**Supplemental Figure 2** All variables selected from perioperative dataset.


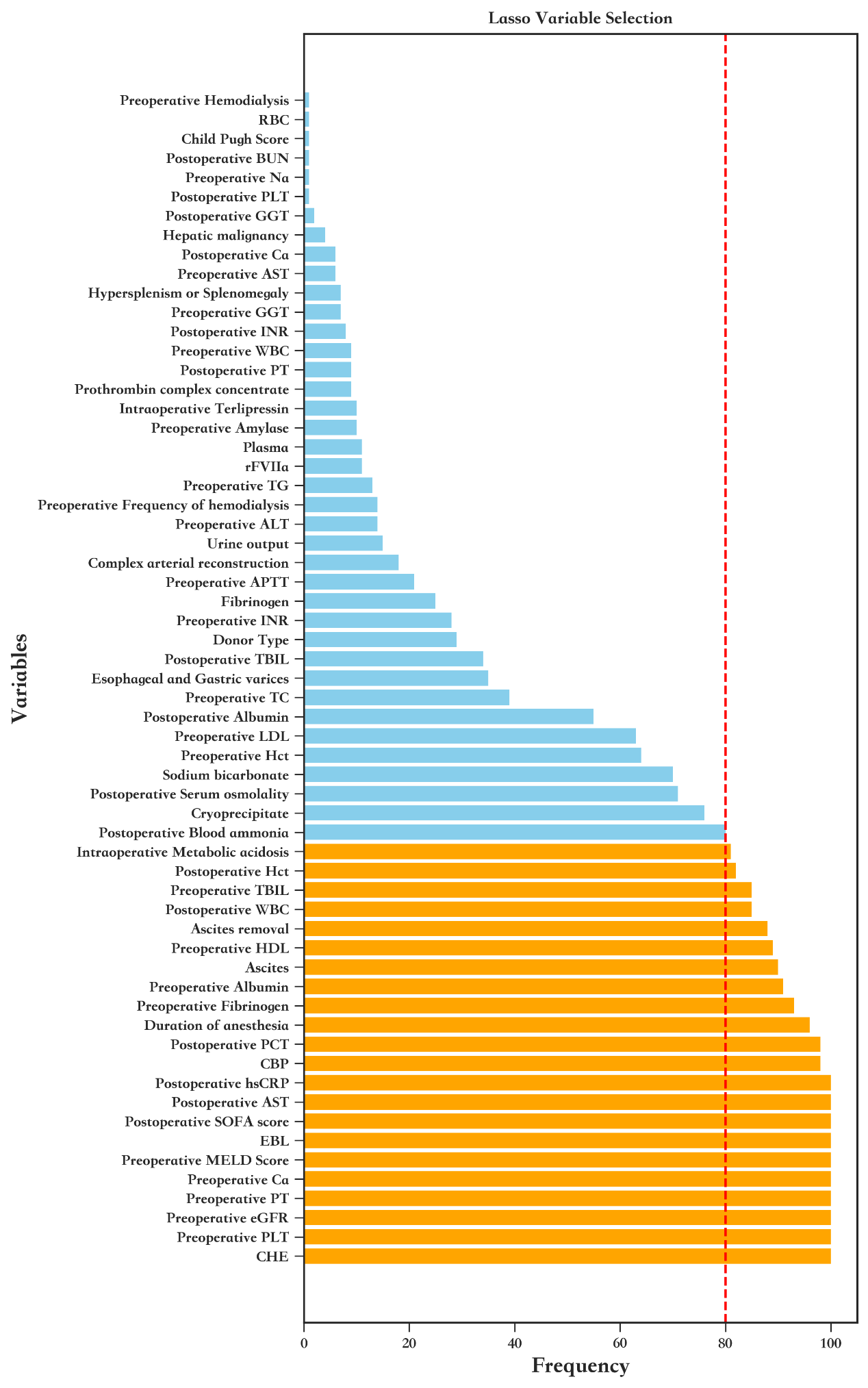


**Supplemental Figure 3** Examples of SHAP decision plot and force plot.


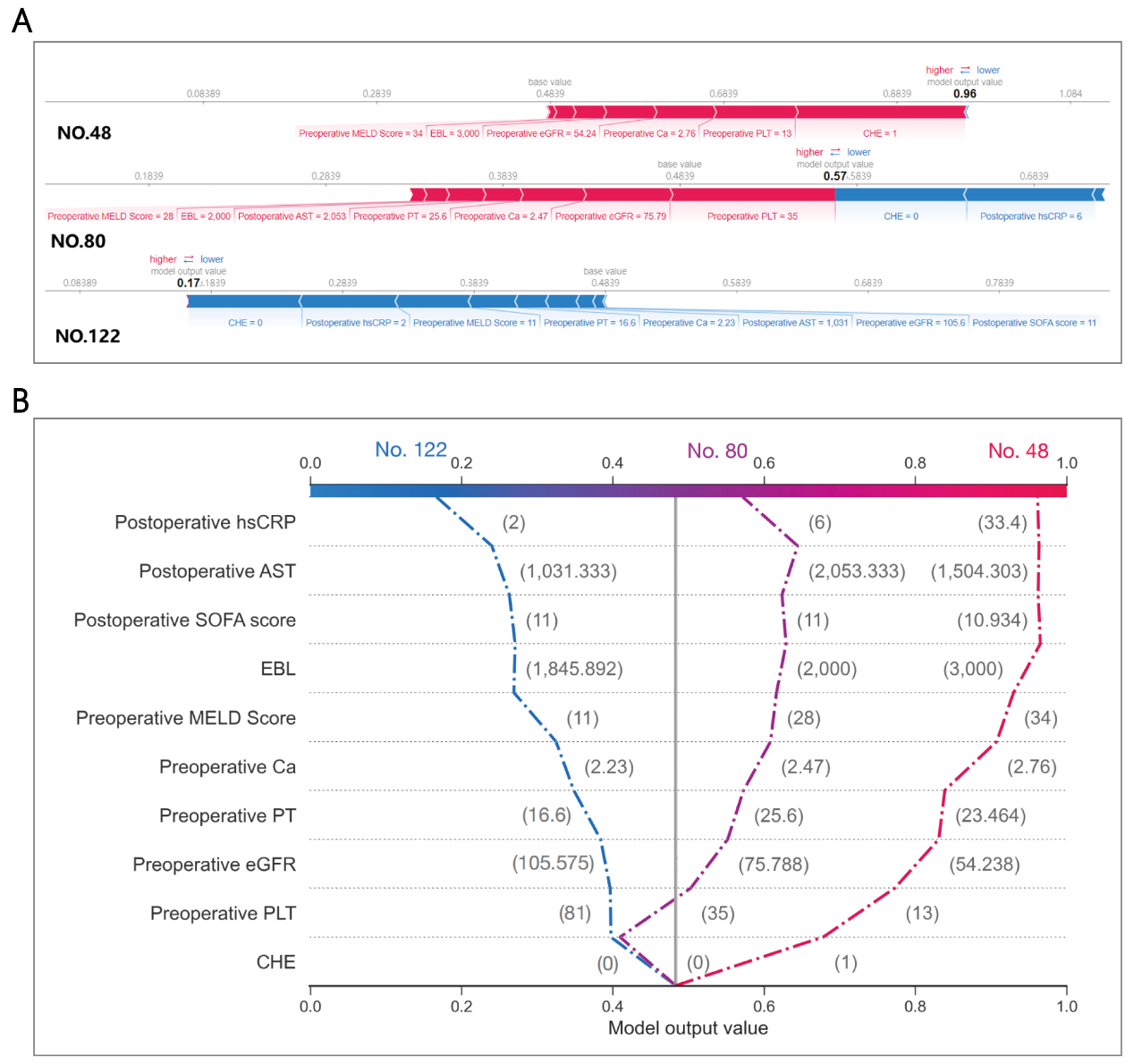


**Supplemental Table 1** A summary of the new nomenclature of perioperative neurocognitive impairment [1, 2].

| **Terms** | **Period** |
| --- | --- |
| Neurocognitive disorder (mild or major [e.g., dementia]) | Preexisting/preoperative cognitive impairment or cognitive impairment developing after 12 months of surgery. |
| Emergence delirium | Delirium diagnosed within minutes or hours after surgery. |
| Postoperative delirium | Delirium diagnosed within days after surgery, up to 1 week or until discharge. |
| Delayed neurocognitive recovery | Cognitive decline up to 30 days postoperatively. |
| Postoperative (neuro)cognitive dysfunction | Cognitive impairment detected between 30 days and 12 months postoperatively. |

**Supplemental Table 2** All variables selected for initial selection.

| **Demographic characteristics** | PAH (n) | **Etiology for liver transplantation** |
| --- | --- | --- |
| Age (y) | Pulmonary nodules (n) | Hepatitis B (n) |
| Sex | Pleural effusion (n) | Hepatitis C (n) |
| Female | AKI (n) | Dual infection (n) |
| Male | CKD (n) | Hepatic malignancy (n) |
| Height (cm) |  | Alcohol liver disease(n) |
|  |  |  |
| Weight (kg) | **Preoperative laboratory results** | Cholestatic cirrhosis (n) |
| BMI | Hemoglobin (g/L) |  |
| Blood group | Hct | **Preoperative complications and treatments** |
|  | WBC (10^9^/L) | Child Pugh Score |
| **Donor characteristics** | Lymphocytes (10^9^/L) | MELD Score |
| Donor age (y) | PLT (10^9^/L) | Hepatorenal syndrome (n) |
| Donor BMI | ALT (U/L) | Hepatopulmonary syndrome (n) |
| Donor Type | AST (U/L) | Ascites (n) |
| Steatosis of donor liver | GGT (g/L) | Esophageal-gastro varices (n) |
|  | Albumin (g/L) | Hypersplenism or Splenomegaly (n) |
| **Preoperative comorbidities** | TBIL (μmol/L) | Portal vein thrombosis (n) |
| ABO incompatibility | IBIL (μmol/L) | Portal hypertension (n) |
| Smoking (n) | eGFR [mL/(min*1.73^2^)] | Spontaneous bacterial peritonitis (n) |
| Alcohol (n) | SCr (μmol/L) | Cirrhosis (n) |
| Drug Abuse (n) | BUN (mmol/L) | Primary biliary cirrhosis (n) |
| History of previous surgery (n) | APTT (s) | Alcoholic cirrhosis (n) |
| Fever (n) | PT (s) | Emergency surgery (n) |
| Stroke (n) | FIB (g/L) | Hemodialysis (n) |
| Cerebrovascular disease (n) | INR | CBP (n) |
| CHE (n) | TG (mmol/L) | PE (n) |
| Diabetes (n) | TC (mmol/L) | MV (n) |
| Hypertension (n) | HDL (mmol/L) | Tracheal intubation (n) |
| CAD (n) | LDL (mmol/L) | Preoperative length of stay (d) |
| Cardiovascular disease (n) | Amylase (U/L) | Preoperative ICU stay (n) |
| COPD (n) | Glu (mmol/L) | Hyponatremia (n) |
| Obsolete tuberculosis (n) | Ca^2+^ (mmol/L) | Hypernatremia (n) |
| Bronchiectasis (n) | Na^+^ (mmol/L) | Hypokalemia (n) |
| ARDS (n) | K^+^ (mmol/L) | Hyperkalemia (n) |
| Primary biliary cirrhosis (n) | HCO3^-^ (mmol/L) | Hypercalcemia (n) |
|  | TBIL (μmol/L) | Metabolic acidosis (n) |
| **Surgery characteristics** | Use of norepinephrine-continuous (n) | INR |
| Day or night surgery | Dopamine-bolus (mg) | hsCRP (mg/L) |
| Duration of surgery (min) | Use of dopamine-continuous (n) | PCT (ng/mL) |
| Duration of anesthesia (min) | Epinephrine bolus (mg) | Ammonia (μmol/L) |
| Duration of cold ischemic (min) | Use of epinephrine-continuous (n) | Serum osmolality (mOsm/(kg·H_2_O)) |
| Duration anhepatic phase (min) | Metaraminol bolus (n) | Glu (mmol/L) |
|  | Dopamine-bolus (mg) | Ca^2+^ (mmol/L) |
| **Surgical technique** |  | Na^+^ (mmol/L) |
| Piggyback (n) | **Intra-operative incident** | K^+^ (mmol/L) |
| Split liver (n) | Hypokalemia (n) |  |
| Standard (n) | Hypernatremia (n) |  |
| Biliary intestinal anastomosis (n) | Metabolic acidosis (n) |  |
| Complex arterial reconstruction (n) | Hyperlactatemia (n) |  |
|  | Cardiac arrhythmia (n) |  |
| **Intra-operative fluid and transfusion** | Cardiac arrest (n) |  |
| Crystalloid (mL) | Cardiac arrhythmia (n) |  |
| Colloid (mL) | Hypotension (n) |  |
| Sodium bicarbonate (mL) |  |  |
| Albumin (mL) | **Postoperative relative scores** |  |
| RBC (mL) | MELD score |  |
| Plasma (mL) | SOFA score |  |
| Cryoprecipitate (U) |  |  |
| Other fluid infusion (mL) | **Postoperative laboratory results** | |
| Total volume of fluid infusion (mL) | Hct |  |
| EBL (mL) | Hemoglobin (g/L) |  |
| Urine output (mL) | WBC (10^9^/L) |  |
| Ascites removal (mL) | Lymphocyte (10^9^/L) |  |
| Gastric drainage (mL) | PLT (10^9^/L) |  |
| Other estimated fluid loss (mL) | ALT (U/L) |  |
| Total volume of fluid loss (mL) | AST (U/L) |  |
| **Intra-operative medications** | GGT (U/L) |  |
| rFVIIa (mg) | Albumin (g/L) |  |
| Prothrombin complex concentrate (IU) | TBIL (μmol/L) |  |
| Fibrinogen (g) | SCr (μmol/L) |  |
| Terlipressin (mg) | BUN (mmol/L) |  |
| Tacrolimus (n) | PT (s) |  |
| Norepinephrine bolus (mg) |  |  |

Abbreviations: BMI, body mass index; DBD, donation after brain death; DCD, donation after circulatory death; DBCD, donation after brain death followed by circulatory death; ASA, American Society of Anesthesiologists; CHE, cover hepatic encephalopathy; CAD, coronary artery disease; COPD, chronic obstructive pulmonary disease; ARDS, acute respiratory distress syndrome; PAH, pulmonary arterial hypertension; AKI, acute kidney injury; CKD, chronic kidney disease; Hct, Hematocrit; WBC, white blood cell; PLT, platelets; ALT, alanine aminotransferase; AST, Aspartate aminotransferase; GGT, gamma-glutamyltransferase; TBIL, total bilirubin; IBIL, indirect bilirubin; eGFR, estimated glomerular filtration rate; SCr, serum creatinine; BUN, blood urea nitrogen; APTT, activated partial thromboplastin time; PT, prothrombin time; FIB, fibrinogen; INR, international normalized ratio; TG, triglyceride; TC, total cholesterol; HDL, high density lipoprotein; LDL, low density lipoprotein; Glu, glucose; MELD scores, model for end-stage liver disease score; CBP, continuous blood purification; PE, plasma exchange; MV, mechanical ventilation; ICU, intensive care unit; RBC, red blood cell; EBL, estimated blood loss; rFVIIa, recombinant activated factor VII; SOFA scores, sequential organ failure assessment score; hsCRP, hypersensitive C-reactive protein; PCT, procalcitonin.

**Supplemental Table 3** A summary of the definition of the main complications or relative terms in our study according to our previous study [3-5].

| **Variables** | **Definition** |
| --- | --- |
| ARDS | ARDS was diagnosed when all of the following conditions were met:  1. PaO_2_/FiO_2_ ratio below 200;  2.Pulmonary infiltrates demonstrated by chest X-rays or thoracic spiral CT;  3. The term “ARDS” was extracted by NLP module from admission diagnosis. |
| ALI | ALI was diagnosed when all of the following conditions were met:  1. PaO_2_/FiO_2_ ratio below 300;  2. Pulmonary infiltrates demonstrated by chest X-rays or thoracic spiral CT. |
| AKI | The ratio of preoperative maximum SCr to minimum SCr was over 1.5. |
| CKD | Identified eGFR below 60 for 3 months or more or identified markedly high levels of albumin in urine test. |
| Sepsis | Identified as an acute change in total SOFA score ≥ 2 points consequent to related infection. |
| Pneumonia | At least one of the following definitive chest X-ray or CT findings was fulfilled: infiltrate, consolidation, cavitation; and at least one of the following signs and symptoms of infection (Temperature>38 °C or<36 °C with no other causes, white blood cell (WBC) count>10× 10^9^/L or <4× 10^9^/L) |
| Recipient warm ischemia time | The time of liver implantation, from removal of the organ from ice until reperfusion. |
| Hypokalemia | Serum potassium was lower than 3.5 mmol/L. |
| Hyperkalemia | Serum potassium was higher than 5.5 mmol/L. |
| Hyponatremia | Serum sodium was lower than 135 mmol/L. |
| Hypernatremia | Serum sodium was higher than 145 mmol/L. |
| Hypocalcemia | Ionized calcium was lower than 1.0 mmol/L. |
| Hypercalcemia | Ionized calcium was higher than 2.75 mmol/L. |
| Metabolic acidosis | HCO_3_^-^ was below 20.2 mmol/L. |
| Arrhythmia | Arrhythmia was diagnosed when any of the criteria was met:  1. Detection of any intraoperative heart rate over 100 bpm or below 50 bpm;  2. Intraoperative use of any of the following antiarrhythmic drugs including amiodarone, lidocaine, lanatoside D, epinephrine, atropine or isoprenaline was identified. |
| Cardiac arrest | The term “cardiac arrest” was identified by NLP module from surgical or anesthesia record. |
| Acidosis | pH in blood gas result was lower than 7.30. |
| Hyperlactacidemia | Serum lactate was over 2.0 mmol/L. |
| Intraoperative hypotension | Hypotension was diagnosed when any intraoperative invasive or non-invasive systolic pressure below 90 mmHg or any invasive or non-invasive mean arterial pressure was below 60 mmHg. |
| Reoperation | Occurrences of related remedial procedures resulting from liver transplantation, including but not limited to: re-transplantation, emergency exploratory laparotomy. |

Abbreviations: ARDS = acute respiratory distress syndrome; ALI = acute lung injury; AKI = acute kidney injury; CKD = chronic kidney disease.

**Supplemental Table 4** All variables selected by univariate test.

| **Donor characteristics** | FIB (g/L) | **Surgery characteristics** |
| --- | --- | --- |
| Donor Type | INR | Duration of anesthesia (min) |
|  | TG (mmol/L) | Biliary intestinal anastomosis (n) |
| **Preoperative comorbidities** | TC (mmol/L) | Complex arterial reconstruction (n) |
| ASA classification | HDL (mmol/L) |  |
| ABO incompatibility | LDL (mmol/L) | **Intra-operative fluid and transfusion** |
| Alcohol (n) | Amylase (U/L) | Sodium bicarbonate (mL) |
| Fever (n) | Ca^2+^ (mmol/L) | RBC (mL) |
| CHE (n) | Na^+^ (mmol/L) | Plasma (mL) |
| CAD (n) |  | Cryoprecipitate (U) |
| ARDS (n) | **Etiology for liver transplantation** | Total volume of fluid infusion (mL) |
| PAH (n) | Hepatic malignancy (n) | EBL (mL) |
| Pulmonary nodules (n) |  | Urine output (mL) |
| AKI (n) | **Preoperative Complications and treatments** | Ascites removal (mL) |
| CKD (n) | Child Pugh Score | Gastric drainage (mL) |
| Donor age (y) | MELD Score | Total volume of fluid loss (mL) |
| Donor BMI | Hepatorenal syndrome (n) |  |
|  | Ascites (n) | **Intra-operative medications** |
| **Preoperative laboratory results** | Esophageal-gastro varices (n) | rFVIIa (mg) |
| Hemoglobin (g/L) | Hypersplenism or Splenomegaly (n) | Prothrombin complex concentrate (IU) |
| Hct | Portal hypertension (n) | Fibrinogen (g) |
| WBC (10^9^/L) | Spontaneous bacterial peritonitis (n) | Terlipressin (mg) |
| PLT (10^9^/L) | Cirrhosis (n) |  |
| ALT (U/L) | Hemodialysis (n) | **Intraoperative incident** |
| AST (U/L) | CBP (n) | Metabolic acidosis (n) |
| GGT (g/L) | PE (n) |  |
| Albumin (g/L) | MV (n) | **Intraoperative medication** |
| TBIL (μmol/L) | Tracheal intubation (n) | rFVIIa (mg) |
| IBIL (μmol/L) | Preoperative ICU stay (n) | Prothrombin complex concentrate (IU) |
| eGFR [mL/(min*1.73^2^)] | Hyponatremia (n) | Fibrinogen (g) |
| APTT (s) | Hypernatremia (n) | Terlipressin (mg) |
| PT (s) | Hypercalcemia (n) |  |
| Primary biliary cirrhosis (n) |  |  |
| **Postoperative laboratory results** | TBIL (μmol/L) | Serum osmolality (mOsm/(kg·H_2_O)) |
| Hct | BUN (mmol/L) |  |
| Hemoglobin (g/L) | PT (s) | **Postoperative relative scores** |
| WBC (10^9^/L) | INR | MELD score |
| PLT (10^9^/L) | hsCRP (mg/L) | SOFA score |
| AST (U/L) | PCT (ng/mL) |  |
| GGT (U/L) | Ammonia (μmol/L) |  |

Abbreviations: ASA, American Society of Anesthesiologists; CHE, cover hepatic encephalopathy; CAD, coronary artery disease; ARDS, acute respiratory distress syndrome; PAH, pulmonary arterial hypertension; AKI, acute kidney injury; CKD, chronic kidney disease; Hct, Hematocrit; WBC, white blood cell; PLT, platelets; ALT, alanine aminotransferase; AST, Aspartate aminotransferase; GGT, gamma-glutamyltransferase; TBIL, total bilirubin; IBIL, indirect bilirubin; eGFR, estimated glomerular filtration rate; APTT, activated partial thromboplastin time; PT, prothrombin time; FIB, fibrinogen; INR, international normalized ratio; TG, triglyceride; TC, total cholesterol; HDL, high density lipoprotein; LDL, low density lipoprotein; MELD scores, model for end-stage liver disease score; CBP, continuous blood purification; PE, plasma exchange; MV, mechanical ventilation; ICU, intensive care unit; RBC, red blood cell; EBL, estimated blood loss; rFVIIa, recombinant activated factor VII; hsCRP, hypersensitive C-reactive protein

**Supplemental Table 5** Details of the optimal hyperparameters for each classifier.

|  | **Hyperparameter set** | **Optimal hyperparameters** | **AUC** |
| --- | --- | --- | --- |
| LR | penalty: ['l2', 'l1','elasticnet', 'none']  C: [0.01, 0.1, 1, 10]  solver: ['newton-cg', 'lbfgs', 'liblinear', 'sag', 'saga']  max_iter: [10,30,50] | penalty='none'  C=0.01  solver='newton-cg'  max_iter=10 | 0.799 |
| MLP | hidden_layer_sizes: [ (32,32), (64, 64)]  activation: ['relu','identity']  solver: [ 'sgd', 'adam']  alpha: [ 0.001,0.01]  learning_rate_init: [ 0.01,0.1] | hidden_layer_sizes=(32, 32)  activation='identity'  solver='sgd'  alpha=0.001  learning_rate_init=0.01 | 0.797 |
| SVM | kernel: ['linear', 'rbf']  C: [0.01, 0.1, 1, 10] | kernel='linear'  C=10 | 0.787 |
| RF | criterion: ["gini", "entropy"]  max_depth: [3, 5,7, 10]  min_samples_leaf: [1, 2, 4]  n_estimators: [20,50,100] | criteria = 'gini'  max_depth=5  min_samples_leaf=2  n_estimators=50 | 0.791 |
| LGB | max_depth: [3,5,7]  num_leaves: [10,20, 40]  learning_rate: [ 0.01, 0.1, 1]  num_iterations: [50,100,200] | max_depth=7  num_leaves=10  learning_rate=0.01  num_iterations=200 | 0.792 |
| XGB | max_depth: [3, 5, 7]  lambda: [0.01, 0.1, 1, 10]  learning_rate: [0.1, 0.2, 0.3] | max_depth=5  lambda=10  learning_rate=0.1 | 0.795 |

Note: AUC, area under the curve of ROC; LR, logistic regression; MLP, multi-layer perceptron classifier; SVM, support vector machine; RF, random forest; LGB, light gradient boosting machine, XGB, extreme gradient boosting with classification trees.

**Supplemental Table 6.** Perioperative variables of patients with stratification by perioperative neurocognitive disorder.

| Characteristics | | | All (n=600) | | NonPND (n=399) | | | | PND (n=201) | | *P* value | | |
| --- | --- | --- | --- | --- | --- | --- | --- | --- | --- | --- | --- | --- | --- |
| **Pre-operative comorbidities** | | | | | | | | | | | | | |
|  | ASA classification | |  | |  | | | |  | | **<0.001** | | |
|  | Ⅱ | | 55(9.17%) | | 49(14.24%) | | | | 6(3.95%) | |  | | |
|  | Ⅲ | | 441(73.50%) | | 295(85.76%) | | | | 146(96.05%) | |  | | |
|  | ABO incompatibility | | 74(12.33%) | | 41(11.17%) | | | | 33(17.84%) | | **0.03** | | |
|  | Smoking (n) | | 190(31.67%) | | 126(35.10%) | | | | 64(35.56%) | | 0.916 | | |
|  | Alcohol (n) | | 145(24.17%) | | 87(24.23%) | | | | 58(32.22%) | | **0.049** | | |
|  | Drug Abuse (n) | | 4(0.67%) | | 1(0.25%) | | | | 3(1.49%) | | 0.078 | | |
|  | History of previous surgery (n) | | 40(6.67%) | | 21(5.72%) | | | | 19(10.27%) | | 0.052 | | |
|  | Fever (n) | | 62(10.33%) | | 34(8.52%) | | | | 28(13.93%) | | **0.04** | | |
|  | Stroke (n) | | 6(1.00%) | | 5(1.25%) | | | | 1(0.50%) | | 0.38 | | |
|  | Cerebrovascular disease (n) | | 4(0.67%) | | 1(0.28%) | | | | 3(1.67%) | | 0.077 | | |
|  | CHE (n) | | 126(21.00%) | | 35(8.77%) | | | | 91(45.27%) | | **<0.001** | | |
|  | Diabetes (n) | | 84(14.00%) | | 56(15.60%) | | | | 28(15.56%) | | 0.99 | | |
|  | Hypertension (n) | | 53(8.83%) | | 34(9.50%) | | | | 19(10.56%) | | 0.698 | | |
|  | CAD (n) | | 6(1.00%) | | 1(0.27%) | | | | 5(2.70%) | | **0.009** | | |
|  | Cardiovascular disease (n) | | 22(3.67%) | | 15(4.18%) | | | | 7(3.89%) | | 0.873 | | |
|  | COPD (n) | | 24(4.00%) | | 16(4.46%) | | | | 8(4.44%) | | 0.995 | | |
|  | Obsolete tuberculosis (n) | | 16(2.67%) | | 11(3.06%) | | | | 5(2.78%) | | 0.853 | | |
|  | Bronchiectasis (n) | | 13(2.17%) | | 8(2.23%) | | | | 5(2.78%) | | 0.695 | | |
|  | ARDS (n) | | 148(24.67%) | | 63(17.55%) | | | | 85(47.22%) | | **<0.001** | | |
|  | PAH (n) | | 3(0.50%) | | 0(0%) | | | | 3(1.67%) | | **0.014** | | |
|  | Pulmonary nodules (n) | | 161(26.83%) | | 118(32.87%) | | | | 43(23.89%) | | **0.032** | | |
|  | Pleural effusion (n) | | 141(23.50%) | | 96(26.74%) | | | | 45(25.00%) | | 0.664 | | |
|  | AKI (n) | | 113(18.83%) | | 58(15.80%) | | | | 55(29.73%) | | **<0.001** | | |
|  | CKD (n) | | 79(13.17%) | | 40(10.90%) | | | | 39(21.08%) | | **<0.001** | | |
| **Pre-operative laboratory results** | | | | | | | | | | | |  |  |
|  | Hemoglobin (g/L) | 100.00 (122.00-82.50) | | | 104.00 (126.50-84.00) | | 94.00 (116.00-80.75) | | | **<0.001** | |  |  |
|  | Hct | 0.29 (0.36-0.24) | | | 0.30 (0.37-0.25) | | 0.27 (0.33-0.22) | | | **<0.001** | |  |  |
|  | WBC (10^9^/L) | 5.27 (8.51-3.68) | | | 5.04 (7.30-3.60) | | 6.11 (10.71-3.81) | | | **<0.001** | |  |  |
|  | Lymphocytes (10^9^/L) | 0.90 (1.32-0.61) | | | 0.91 (1.40-0.62) | | 0.86 (1.25-0.58) | | | 0.224 | |  |  |
|  | PLT (10^9^/L) | 74.00 (127.25-49.00) | | | 88.00 (151.00-56.00) | | 58.00 (89.00-42.00) | | | **<0.001** | |  |  |
|  | ALT (U/L) | 36.50 (66.25-23.00) | | | 34.00 (58.75-22.00) | | 48.00 (95.50-24.00) | | | **0.003** | |  |  |
|  | AST (U/L) | 60.50 (109.25-36.00) | | | 51.50 (97.00-33.00) | | 86.00 (132.00-47.25) | | | **<0.001** | |  |  |
|  | GGT (g/L) | 51.00 (108.00-30.00) | | | 57.00 (125.25-32.00) | | 43.00 (71.00-26.00) | | | **<0.001** | |  |  |
|  | Albumin (g/L) | 35.30 (38.85-32.40) | | | 35.70 (39.30-32.30) | | 34.90 (38.30-32.50) | | | **0.027** | |  |  |
|  | TBIL (μmol/L) | 96.78 (433.69-22.77) | | | 40.21 (246.84-17.26) | | 365.26 (543.22-139.90) | | | **<0.001** | |  |  |
|  | IBIL (μmol/L) | 29.65 (131.97-9.80) | | | 15.30 (76.40-8.25) | | 112.20 (194.30-42.90) | | | **<0.001** | |  |  |
|  | eGFR [mL/(min*1.73^2^)] | 101.74 (115.44-77.56) | | | 102.34 (115.29-82.19) | | 99.15 (116.50-66.36) | | | **0.036** | |  |  |
|  | SCr (μmol/L) | 75.00 (94.00-61.00) | | | 74.00 (89.50-62.00) | | 76.00 (110.00-59.00) | | | 0.211 | |  |  |
|  | BUN (mmol/L) | 4.60 (6.92-3.52) | | | 4.46 (6.42-3.51) | | 5.13 (7.54-3.55) | | | 0.135 | |  |  |
|  | APTT (s) | 47.80 (61.98-39.70) | | | 43.90 (55.20-38.65) | | 56.70 (71.30-47.50) | | | **<0.001** | |  |  |
|  | PT (s) | 18.80 (28.30-14.90) | | | 16.90 (22.88-14.40) | | 27.80 (36.55-18.15) | | | **<0.001** | |  |  |
|  | FIB (g/L) | 1.69 (2.73-1.07) | | | 2.10 (3.07-1.27) | | 1.18 (1.85-0.93) | | | **<0.001** | |  |  |
|  | INR | 1.66 (2.80-1.21) | | | 1.37 (2.20-1.13) | | 2.60 (3.71-1.67) | | | **<0.001** | |  |  |
|  | TG (mmol/L) | 0.75 (1.06-0.55) | | | 0.78 (1.12-0.56) | | 0.69 (0.97-0.53) | | | **0.012** | |  |  |
|  | TC (mmol/L) | 3.05 (3.93-2.11) | | | 3.25 (4.19-2.39) | | 2.49 (3.42-1.76) | | | **<0.001** | |  |  |
|  | HDL (mmol/L) | 0.49 (0.93-0.16) | | | 0.64 (1.01-0.23) | | 0.21 (0.58-0.11) | | | **<0.001** | |  |  |
|  | LDL (mmol/L) | 1.55 (2.31-0.97) | | | 1.73 (2.54-1.14) | | 1.20 (1.79-0.74) | | | **<0.001** | |  |  |
|  | Amylase (U/L) | 75.00 (106.25-54.00) | | | 73.00 (100.00-52.00) | | 79.00 (119.00-55.50) | | | **0.017** | |  |  |
|  | Glu (mmol/L) | 4.93 (6.25-4.18) | | | 4.83 (5.81-4.26) | | 5.22 (7.21-4.02) | | | 0.176 | |  |  |
|  | Ca^2+^ (mmol/L) | 2.30 (2.45-2.17) | | | 2.28 (2.41-2.16) | | 2.37 (2.50-2.19) | | | **<0.001** | |  |  |
|  | Na^+^ (mmol/L) | 139.10 (142.00-135.50) | | | 139.70 (142.10-136.00) | | 138.30 (141.50-134.20) | | | **0.01** | |  |  |
|  | K^+^ (mmol/L) | 3.84 (4.14-3.53) | | | 3.86 (4.14-3.55) | | 3.80 (4.11-3.43) | | | 0.206 | |  |  |
|  | HCO3^-^ (mmol/L) | 22.70 (24.70-20.30) | | | 22.50 (24.60-20.60) | | 22.90 (25.10-19.80) | | | 0.806 | |  |  |
| **Etiology for liver transplantation** | | | | | | | | | | | |  |  |
|  | Hepatitis B (n) | 417(69.50%) | | | 274(74.66%) | | 143(77.30%) | | | 0.496 | |  |  |
|  | Hepatitis C (n) | 14(2.33%) | | | 11(3.00%) | | 3(1.62%) | | | 0.332 | |  |  |
|  | Dual infection (n) | 7(1.17%) | | | 5(1.36%) | | 2(1.08%) | | | 0.78 | |  |  |
|  | Hepatic malignancy (n) | 263(43.83%) | | | 218(55.19%) | | 45(22.50%) | | | **<0.001** | |  |  |
|  | Alcohol liver disease(n) | 30(5.00%) | | | 16(4.36%) | | 14(7.57%) | | | 0.117 | |  |  |
|  | Cholestatic cirrhosis (n) | 14(2.33%) | | | 11(3.16%) | | 3(1.96%) | | | 0.453 | |  |  |
| **Pre-operative complications and treatments** | | | | | | | | | | | |  |  |
|  | Child Pugh Score | 10.00 (11.00-7.00) | | | 9.00 (10.00-6.00) | | 10.00 (11.00-9.00) | | | **<0.001** | |  |  |
|  | MELD Score | 22.00 (34.20-22.00) | | | 22.00 (27.00-22.00) | | 33.40 (40.00-25.00) | | | **<0.001** | |  |  |
|  | Hepatorenal syndrome (n) | 18(3.00%) | | | 5(1.40%) | | 13(7.22%) | | | **<0.001** | |  |  |
|  | Hepatopulmonary syndrome (n) | 2(0.33%) | | | 1(0.27%) | | 1(0.54%) | | | 0.621 | |  |  |
|  | Ascites (n) | 238(39.67%) | | | 171(46.59%) | | 67(36.22%) | | | **0.02** | |  |  |
|  | Esophageal-gastro varices (n) | 327(54.50%) | | | 234(65.18%) | | 93(51.67%) | | | **0.002** | |  |  |
|  | Hypersplenism or Splenomegaly (n) | 333(55.50%) | | | 238(59.65%) | | 95(47.26%) | | | **0.004** | |  |  |
|  | Portal vein thrombosis (n) | 34(5.67%) | | | 27(7.54%) | | 7(3.89%) | | | 0.1 | |  |  |
|  | Portal hypertension (n) | 404(67.33%) | | | 279(77.72%) | | 125(69.44%) | | | **0.037** | |  |  |
|  | Spontaneous bacterial peritonitis (n) | 49(8.17%) | | | 20(5.57%) | | 29(16.11%) | | | **<0.001** | |  |  |
|  | Cirrhosis (n) | 460(76.67%) | | | 316(86.10%) | | 144(77.84%) | | | **0.014** | |  |  |
|  | Primary biliary cirrhosis (n) | 6(1.00%) | | | 3(0.82%) | | 3(1.62%) | | | 0.39 | |  |  |
|  | Alcoholic cirrhosis (n) | 20(3.33%) | | | 13(3.54%) | | 7(3.78%) | | | 0.886 | |  |  |
|  | Emergency surgery (n) | 124(20.67%) | | | 80(20.51%) | 44(22.00%) | | | | 0.675 | |  |  |
|  | Hemodialysis (n) | 53(8.83%) | | | 20(5.45%) | 33(17.84%) | | | | **<0.001** | |  |  |
|  | CBP (n) | 158(26.33%) | | | 63(17.45%) | 95(52.20%) | | | | **<0.001** | |  |  |
|  | PE (n) | 27(4.50%) | | | 9(2.51%) | 18(10.00%) | | | | **<0.001** | |  |  |
|  | MV (n) | 16(2.67%) | | | 1(0.28%) | 15(8.33%) | | | | **<0.001** | |  |  |
|  | Tracheal intubation (n) | 21(3.50%) | | | 6(1.63%) | 15(8.11%) | | | | **<0.001** | |  |  |
|  | Preoperative length of stay (d) | 11.00 (26.00-2.00) | | | 12.00 (26.50-2.00) | 10.00 (24.25-2.75) | | | | 0.775 | |  |  |
|  | Preoperative ICU stay (n) | 249(41.50%) | | | 154(44.25%) | 95(62.09%) | | | | **<0.001** | |  |  |
|  | Hyponatremia (n) | 122(20.33%) | | | 67(18.26%) | 55(29.73%) | | | | **0.002** | |  |  |
|  | Hypernatremia (n) | 29(4.83%) | | | 12(3.27%) | 17(9.19%) | | | | **0.003** | |  |  |
|  | Hypokalemia (n) | 132(22.00%) | | | 79(21.53%) | 53(28.65%) | | | | 0.064 | |  |  |
|  | Hyperkalemia (n) | 1(0.17%) | | | 1(0.27%) | 0(0%) | | | | 0.477 | |  |  |
|  | Hypercalcemia (n) | 19(3.17%) | | | 5(1.36%) | 14(7.57%) | | | | **<0.001** | |  |  |
|  | Metabolic acidosis (n) | 238(39.67%) | | | 160(43.60%) | 78(42.16%) | | | | 0.748 | |  |  |
| **Intra-operative surgery Characteristics** | | | | | | | | | | | | |  |
| Day or night surgery | | |  |  | | | |  | | 0.438 | | |  |
|  | Day (n) | | 383(63.83%) | 259(64.91%) | | | | 124(61.69%) | |  | | |  |
|  | Night (n) | | 217(36.17%) | 140(35.09%) | | | | 77(38.31%) | |  | | |  |
|  | Duration of surgery (min) | | 430.00 (482.00-379.00) | 425.00 (480.00-370.00) | | | | 440.00 (488.00-390.00) | | 0.078 | | |  |
|  | Duration of anesthesia(min) | | 520.00 (585.00-470.00) | 510.00 (576.50-460.00) | | | | 537.00 (596.25-490.00) | | **<0.001** | | |  |
|  | Duration of cold ischemic (min) | | 360.00 (410.00-330.00) | 360.00 (410.00-330.00) | | | | 360.00 (412.50-330.00) | | 0.81 | | |  |
|  | Duration of anhepatic phase (min) | | 45.00 (52.00-39.00) | 45.00 (52.00-39.00) | | | | 45.00 (52.25-39.75) | | 0.624 | | |  |
|  | Surgical technique | |  |  | | | |  | | 0.152 | | |  |
|  | Piggyback (n) | | 502(83.67%) | 334(91.01%) | | | | 168(91.30%) | |  | | |  |
|  | Split liver (n) | | 27(4.50%) | 15(4.09%) | | | | 12(6.52%) | |  | | |  |
|  | Standard (n) | | 22(3.67%) | 18(4.90%) | | | | 4(2.17%) | |  | | |  |
|  | Biliary intestinal anastomosis (n) | | 16(2.67%) | 15(4.09%) | | | | 1(0.55%) | | **0.02** | | |  |
|  | Complex arterial reconstruction (n) | | 137(22.83%) | 104(28.34%) | | | | 33(17.93%) | | **0.008** | | |  |
| **Intra-operative fluid and transfusion** | | | | | | | | | | | | |  |
|  | Crystalloid (mL) | | 3253.34(1457.72) | | 3247.66(1412.08) | | | 3264.67(1548.74) | | 0.8 | | |  |
|  | Colloid (mL) | | 130.61(270.19) | | 138.30(274.17) | | | 115.28(262.14) | | 0.334 | | |  |
|  | Sodium bicarbonate (mL) | | 126.47(461.36) | | 114.98(544.70) | | | 149.39(213.46) | | **0.001** | | |  |
|  | Albumin (mL) | | 287.72(449.24) | | 263.20(347.99) | | | 336.61(600.64) | | 0.111 | | |  |
|  | RBC (mL) | | 1250.00 (1800.00-750.00) | | 1000.00 (1550.00-625.00) | | | 1500.00 (2500.00-1000.00) | | **<0.001** | | |  |
|  | Plasma (mL) | | 2400.00 (3100.00-1900.00) | | 2400.00 (3000.00-1800.00) | | | 2700.00 (3425.00-2000.00) | | **<0.001** | | |  |
|  | Cryoprecipitate (U) | | 29.64(15.80) | | 27.32(14.74) | | | 34.23(16.82) | | **<0.001** | | |  |
|  | Other fluid infusion (mL) | | 61.24(302.87) | | 64.90(326.80) | | | 53.85(248.37) | | 0.918 | | |  |
|  | Total volume of fluid infusion (mL) | | 7375.00 (9411.25-6030.00) | | 7095.00 (9010.00-5782.50) | | | 7987.50 (10486.25-6361.25) | | **<0.001** | | |  |
|  | EBL (mL) | | 1200.00(1200.00) | | 1000.00(1200.00) | | | 1700.00(2000.00) | | **<0.001** | | |  |
|  | Urine output (mL) | | 1500.00(1200.00) | | 1600.00(1375.00) | | | 1300.00(1200.00) | | **<0.001** | | |  |
|  | Ascites removal (mL) | | 300.00 (2000.00-0.00) | | 300.00 (1500.00-0.00) | | | 1000.00 (2000.00-0.00) | | **<0.001** | | |  |
|  | Gastric drainage (mL) | | 0.00 (50.00-0.00) | | 0.00 (50.00-0.00) | | | 0.00 (100.00-0.00) | | **0.016** | | |  |
|  | Other estimated fluid loss (mL) | | 300.00 (2000.00-0.00) | | 300.00 (1500.00-0.00) | | | 1000.00 (2000.00-0.00) | | 0.093 | | |  |
|  | Total volume of fluid loss (mL) | | 3867.50 (5642.50-2627.50) | | 3660.00 (5237.50-2501.25) | | | 4275.00 (6125.00-2975.00) | | **0.004** | | |  |
|  | Intra-operative medications | | | |  | | |  | |  | | |  |
|  | rFVIIa (mg) | | 0.28(0.99) | | 0.20(0.89) | | | 0.45(1.16) | | **<0.001** | | |  |
|  | Prothrombin complex concentrate (IU) | | 594.57(430.81) | | 568.39(430.63) | | | 646.49(427.58) | | **0.02** | | |  |
|  | Fibrinogen (g) | | 0.41(1.47) | | 0.41(1.73) | | | 0.42(0.73) | | **0.034** | | |  |
|  | Terlipressin (mg) | | 0.28(0.52) | | 0.22(0.44) | | | 0.40(0.63) | | **0.002** | | |  |
|  | Tacrolimus (n) | | 4(0.67%) | | 2(0.54%) | | | 2(1.08%) | | 0.483 | | |  |
|  | Norepinephrine bolus (mg) | | 0.01(0.02) | | 0.01(0.01) | | | 0.01(0.03) | | 0.883 | | |  |
|  | Use of norepinephrine-continuous (n) | | 464(77.33%) | | 305(83.11%) | | | 159(85.95%) | | 0.39 | | |  |
|  | Dopamine-bolus (mg) | | 0.02(0.19) | | 0.01(0.15) | | | 0.03(0.25) | | 0.208 | | |  |
|  | Use of dopamine-continuous (n) | | 172(28.67%) | | 115(31.34%) | | | 57(30.81%) | | 0.9 | | |  |
|  | Epinephrine bolus (mg) | | 0.03(0.33) | | 0.04(0.36) | | | 0.02(0.26) | | 0.41 | | |  |
|  | Use of epinephrine-continuous (n) | | 395(65.83%) | | 263(71.66%) | | | 132(71.35%) | | 0.939 | | |  |
|  | Metaraminol bolus (n) | | 22(3.67%) | | 12(3.27%) | | | | 10(5.41%) | | 0.226 | | |
|  | Intra-operative incident | |  | |  | | | |  | |  | | |
|  | Hypokalemia (n) | | 220(36.67%) | | 143(38.96%) | | | | 77(41.62%) | | 0.547 | | |
|  | Hypernatremia (n) | | 12(2.00%) | | 8(2.18%) | | | | 4(2.16%) | | 0.989 | | |
|  | Metabolic acidosis (n) | | 224(37.33%) | | 132(35.97%) | | | | 92(49.73%) | | **0.002** | | |
|  | Hyperlactatemia (n) | | 275(45.83%) | | 179(48.77%) | | | | 96(51.89%) | | 0.489 | | |
|  | Cardiac arrhythmia (n) | | 539(89.83%) | | 359(97.82%) | | | | 180(97.30%) | | 0.702 | | |
|  | Cardiac arrest (n) | | 10(1.67%) | | 4(1.09%) | | | | 6(3.24%) | | 0.073 | | |
|  | Cardiac arrhythmia (n) | | 539(89.83%) | | 359(97.82%) | | | | 180(97.30%) | | 0.702 | | |
|  | Hypotension (n) | | 464(77.33%) | | 309(84.20%) | | | | 155(83.78%) | | 0.901 | | |
| **Post-operative assessment** | | | | | | | | | | | | | |
|  | MELD score | | 16.05 (20.12-12.64) | | 14.85 (18.46-11.34) | | | | 19.07 (22.89-15.24) | | **<0.001** | | |
|  | SOFA score | | 10.93(2.71) | | 10.62(2.71) | | | | 11.57(2.63) | | **<0.001** | | |
| **Post-operative laboratory results** | | | | | | | | | | | | | |
|  | Hct | | 0.26 (0.29-0.24) | | 0.27 (0.30-0.24) | | | | 0.25 (0.27-0.22) | | **<0.001** | | |
|  | Hemoglobin (g/L) | | 91.00 (100.00-81.33) | | 92.20 (103.50-82.67) | | | | 87.25 (94.75-78.92) | | **<0.001** | | |
|  | WBC (10^9^/L) | | 8.35 (10.83-5.95) | | 8.49 (11.07-6.16) | | | | 7.62 (10.58-5.46) | | **0.017** | | |
|  | Lymphocyte (10^9^/L) | | 0.30 (0.41-0.21) | | 0.29 (0.40-0.20) | | | | 0.32 (0.45-0.21) | | 0.126 | | |
|  | PLT (10^9^/L) | | 50.00 (82.33-33.00) | | 59.33 (89.67-37.33) | | | | 37.58 (51.42-24.83) | | **<0.001** | | |
|  | ALT (U/L) | | 486.42 (868.83-283.19) | | 483.00 (792.00-275.33) | | | | 496.00 (1020.33-300.33) | | 0.081 | | |
|  | AST (U/L) | | 972.67 (1777.08-554.08) | | 958.00 (1583.00-527.00) | | | | 1098.50 (2222.75-612.33) | | **0.01** | | |
|  | GGT (U/L) | | 55.71 (89.67-37.67) | | 62.00 (100.71-39.33) | | | | 47.50 (66.50-32.88) | | **<0.001** | | |
|  | Albumin (g/L) | | 37.50(4.69) | | 38.10(4.76) | | | | 36.14(4.25) | | **<0.001** | | |
|  | TBIL (μmol/L) | | 77.84 (151.24-43.70) | | 60.44 (113.50-38.71) | | | | 142.52 (213.88-78.11) | | **<0.001** | | |
|  | SCr (μmol/L) | | 94.00 (118.88-78.37) | | 92.67 (116.00-78.00) | | | | 99.00 (127.00-79.00) | | 0.073 | | |
|  | BUN (mmol/L) | | 8.64 (11.29-6.62) | | 7.80 (10.54-6.17) | | | | 10.28 (13.33-8.45) | | **<0.001** | | |
|  | PT (s) | | 16.57 (18.15-15.19) | | 16.34 (17.70-15.00) | | | | 17.38 (18.79-15.65) | | **<0.001** | | |
|  | INR | | 1.34 (1.50-1.20) | | 1.31 (1.45-1.18) | | | | 1.42 (1.56-1.25) | | **<0.001** | | |
|  | hsCRP (mg/L) | | 29.75 (47.45-16.00) | | 28.30 (44.10-15.60) | | | | 32.60 (55.65-18.60) | | **0.013** | | |
|  | PCT (ng/mL) | | 6.58 (20.20-2.54) | | 5.39 (15.26-1.97) | | | | 12.76 (26.90-5.14) | | **<0.001** | | |
|  | Ammonia  (μmol/L) | | 25.80 (35.69-19.46) | | 24.82 (32.36-18.63) | | | | 29.41 (39.89-20.73) | | **<0.001** | | |
|  | Serum osmolality (mOsm/(kg·H_2_O)) | | 314.50 (323.25-307.00) | | 313.00 (320.00-305.00) | | | | 320.00 (331.00-309.00) | | **<0.001** | | |
|  | Glu (mmol/L) | | 13.26 (16.28-10.70) | | 13.04 (16.12-10.67) | | | | 13.66 (16.85-10.78) | | 0.237 | | |
|  | Ca^2+^ (mmol/L) | | 2.52 (2.65-2.42) | | 2.52 (2.62-2.40) | | | | 2.58 (2.73-2.43) | | **0.005** | | |
|  | Na^+^ (mmol/L) | | 142.57 (144.76-140.35) | | 142.30 (144.65-140.15) | | | | 142.95 (145.03-140.87) | | 0.059 | | |
|  | K^+^ (mmol/L) | | 3.76 (4.02-3.53) | | 3.77 (4.02-3.55) | | | | 3.74 (4.00-3.51) | | 0.639 | | |

**Note:** Data were expressed as mean (standard deviation), median (interquartile range) or n (%). Bold data indicates significance at <0.05.

**Abbreviations:** ASA, American Society of Anesthesiologists; CHE, cover hepatic encephalopathy; CAD, coronary artery disease; COPD, chronic obstructive pulmonary disease; ARDS, acute respiratory distress syndrome; PAH, pulmonary arterial hypertension; AKI, acute kidney injury; CKD, chronic kidney disease; Hct, Hematocrit; WBC, white blood cell; PLT, platelets; ALT, alanine aminotransferase; AST, Aspartate aminotransferase; GGT, gamma-glutamyltransferase; TBIL, total bilirubin; IBIL, indirect bilirubin; eGFR, estimated glomerular filtration rate; SCr, serum creatinine; BUN, blood urea nitrogen; APTT, activated partial thromboplastin time; PT, prothrombin time; FIB, fibrinogen; INR, international normalized ratio; TG, triglyceride; TC, total cholesterol; HDL, high density lipoprotein; LDL, low density lipoprotein; Glu, glucose; MELD scores, model for end-stage liver disease score; CBP, continuous blood purification; PE, plasma exchange; MV, mechanical ventilation; ICU, intensive care unit; RBC, red blood cell; EBL, estimated blood loss; rFVIIa, recombinant activated factor VII; MELD scores, model for end-stage liver disease score; SOFA scores, sequential organ failure assessment score; hsCRP, hypersensitive C-reactive protein.

**Supplemental Table 7** Comparison of the demographic characteristics and predicting variables for the development set, internal validation set, temporal external validation set and MIMIC-Ⅳ external validation set.

|  | **Development**  **(N=600)** | | | **Internal**  **(N=151)** | | **Temporal external**  **(N=206)** | | | **MIMIC-Ⅳexternal**  **(N=103)** |
| --- | --- | --- | --- | --- | --- | --- | --- | --- | --- |
| Age (y) | 49.00(10.34) | | | 47.85(11.33) | | 48.37(10.87) | | | 55.00 (61.00-49.00) |
| Sex |  | | |  | |  | | |  |
| Female | 74(12.33%) | | | 23(15.23%) | | 34(16.50%) | | | 26(25.24%) |
| Male | 521(86.83%) | | | 126(83.44%) | | 172(83.50%) | | | 77(74.76%) |
| Height (cm) | 170.00 (172.00-165.00) | | | 170.00 (172.00-165.00) | | 169.00 (172.00-163.25) | | | 172.97(9.03) |
| Weight (kg) | 64.00 (71.00-58.00) | | | 61.00 (70.00-56.00) | | 65.00 (72.00-57.00) | | | 89.55(19.61) |
| Pre-operative predicting variables | | |  | | | |  | | |
| CHE | 126(21.00%) | | | 37(24.50%) | | 47(22.82%) | | | 33(32.04%) |
| MELD Score | 22.00 (34.20-22.00) | | | 26.56(8.17) | | 22.00 (31.00-13.00) | | | 26.00 (33.00-20.00) |
| PLT | 74.00 (127.25-49.00) | | | 61.00 (108.50-39.50) | | 71.00 (131.00-45.00) | | | 86.00 (145.50-59.50) |
| Ca^2+^ | 2.30 (2.45-2.17) | | | 2.32 (2.46-2.19) | | 2.24 (2.40-2.12) | | | 2.20 (2.42-2.01) |
| PT | 18.80 (28.30-14.90) | | | 18.60 (28.90-14.50) | | 21.05 (31.10-16.40) | | | 19.50 (23.25-17.05) |
| eGFR | 101.74 (115.44-77.56) | | | 103.88 (119.49-84.49) | | 103.27 (117.67-85.29) | | | 78.37(40.17) |
| Intra-operative predicting variables | |  | | |  | | |  | |
| EBL | 1200.00 (2000.00-800.00) | | | 1100.00 (2000.00-800.00) | | 1000.00 (1900.00-600.00) | | | 1000.00 (1900.00-600.00) |
| Post-operative predicting variables | | | | |  | | |  | |
| SOFA score | 10.93(2.71) | | | 11.33(2.63) | | 10.00 (13.00-8.00) | | | 10.00 (13.00-8.00) |
| hsCRP | 29.75 (47.45-16.00) | | | 28.60 (46.40-15.80) | | 10.60 (18.80-7.12) | | | 7.70 (31.75-2.85) |
| AST | 972.67 (1777.08-554.08) | | | 953.08 (1905.58-543.00) | | 1094.00 (2178.50-580.25) | | | 694.00 (1714.00-239.00) |

Note: Data were expressed as mean (standard deviation), median (interquartile range) or n (%).

Abbreviations: CHE, cover hepatic encephalopathy; MELD scores, model for end-stage liver disease score; PLT, platelets; PT, prothrombin time; eGFR, estimated glomerular filtration rate ; EBL, estimated blood loss; SOFA scores, sequential organ failure assessment score; hsCRP, hypersensitive C-reactive protein; AST, aspartate aminotransferase.

**Supplemental Table 8**: Comparison of complications and prognosis between Non-PND group and PND group.

|  | **All (N=600)** | **Non-PND (N=399)** | **PND (N=201)** | ***P****-value* |
| --- | --- | --- | --- | --- |
| Sepsis (n) | 154(25.67%) | 75(21.55%) | 79(51.63%) | **<0.001** |
| Pneumonia (n) | 371(61.83%) | 235(65.46%) | 136(75.56%) | **0.017** |
| AKI (n) | 296(49.33%) | 157(39.75%) | 139(69.50%) | **<0.001** |
| Reoperation (n) | 34(5.67%) | 14(3.51%) | 20(9.95%) | **<0.001** |
| Hemodialysis (n) | 142(23.67%) | 47(12.81%) | 95(51.35%) | **<0.001** |
| Frequency of hemodialysis | 0.00 (1.00-0.00) | 0.00 (0.00-0.00) | 2.00 (21.00-0.00) | **<0.001** |
| Hospitalization cost (yuan) | 295928.94(132441.40) | 277018.95(92779.91) | 377801.69(177855.53) | **<0.001** |
| Total length of stay (d) | 39.00 (56.00-26.00) | 38.00 (55.00-25.25) | 41.00 (58.00-27.50) | 0.186 |
| Postoperative length of stay (d) | 22.00(13.00) | 21.00(11.00) | 25.00(18.00) | **<0.001** |
| Postoperative ICU stay (h) | 72.00(63.00) | 65.00(48.50) | 113.00(114.00) | **<0.001** |
| Norepinephrine bolus (mg) | 4.67(11.46) | 4.07(11.25) | 5.86(11.83) | **0.016** |
| Use of norepinephrine (n) | 86(14.33%) | 40(10.90%) | 46(24.86%) | **<0.001** |
| Dopamine bolus (mg) | 43.16(68.45) | 37.22(62.26) | 54.92(78.17) | **0.007** |
| Use of dopamine (n) | 118(19.67%) | 62(16.89%) | 56(30.27%) | **<0.001** |
| Epinephrine bolus (mg) | 1.88(4.30) | 1.89(4.65) | 1.86(3.50) | 0.594 |
| Use of epinephrine (n) | 5(0.83%) | 4(1.09%) | 1(0.54%) | 0.52 |
| Terlipressin (mg) | 0.12(0.36) | 0.07(0.28) | 0.21(0.48) | **<0.001** |
| In-hospital death (n) | 35(5.83%) | 10(2.51%) | 25(12.44%) | **<0.001** |

Note: Data were expressed as mean (standard deviation), median (interquartile range) or n (%). Bold data indicates significance at <0.05.

Abbreviations: AKI, acute kidney injury; ICU, intensive care unit.

**Reference**

1. Tasbihgou SR, Absalom AR, (2021) Postoperative neurocognitive disorders. Korean J Anesthesiol 74: 15-22

2. Evered L, Silbert B, Knopman DS, Scott DA, DeKosky ST, Rasmussen LS, Oh ES, Crosby G, Berger M, Eckenhoff RG, (2018) Recommendations for the nomenclature of cognitive change associated with anaesthesia and surgery-2018. Br J Anaesth 121: 1005-1012

3. Chen C, Chen B, Yang J, Li X, Peng X, Feng Y, Guo R, Zou F, Zhou S, Hei Z, (2023) Development and validation of a practical machine learning model to predict sepsis after liver transplantation. Ann Med 55: 624-633

4. Chen C, Yang D, Gao S, Zhang Y, Chen L, Wang B, Mo Z, Yang Y, Hei Z, Zhou S, (2021) Development and performance assessment of novel machine learning models to predict pneumonia after liver transplantation. Respir Res 22: 94

5. Zhang LM, Hornor MA, Robinson T, Rosenthal RA, Ko CY, Russell MM, (2020) Evaluation of Postoperative Functional Health Status Decline Among Older Adults. JAMA Surg 155: 950-958
